# Supplementary material for: Real-time optical and electronic sensing with a β-amino enone linked, triazine-containing 2D covalent organic framework
Source: Nat Commun. 2019 Jul 19;10:3228. doi: 10.1038/s41467-019-11264-z (PMC6642192; doi:10.1038/s41467-019-11264-z)
Supplement: Supplementary file 1 — Supplementary Information [file 41467_2019_11264_MOESM1_ESM.pdf]

Supporting Information

Real-time optical and electronic sensing with a β-amino enone linked, triazine-containing 2D covalent organic framework

Kulkarni et al.

Supplementary Methods

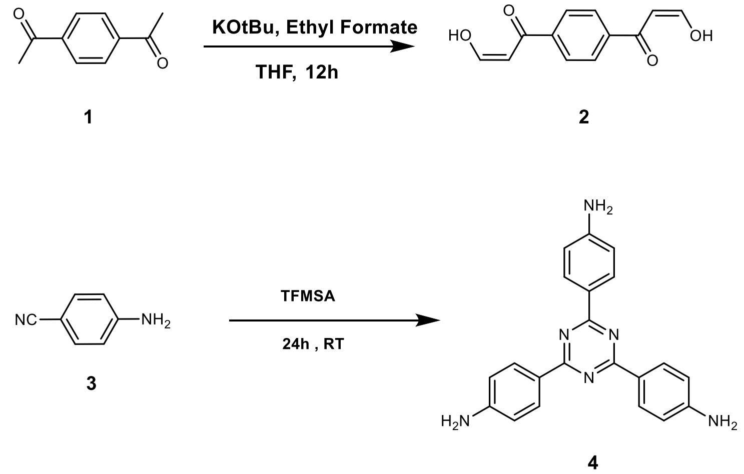

Supplementary Figure 1. Synthesis of PBHP (2) and TAPT (4) monomers

<u>Synthesis of (*2Z,2'Z*)-1,1'-(1,4-phenylene)bis(3-hydroxyprop-2-en-1-one) (2) (PBHP):</u>

A solution of 1,4-diacetylbenzene (1 g, 6.16 mmol) in 80 ml of anhydrous THF was added drop-wise to tBuOK (3.45 g, 30.82 mmol in 100 ml of anhydrous THF) at -78 ºC under argon atmosphere. After stirring at the same temperature for additional 1 hr a light yellow solution was obtained. To this ethyl formate (4.56 g, 4.95 mL, 61.65 mmol) was added and allowed the reaction mixture to warm up to room temperature and continued the stirring for 12 h. The reaction progress was monitored by TLC. After completion the reaction was quenched with 3N HCl (60 ml) and the organic layer were extracted with ethyl acetate (4 x100 mL) dried over $MgSO_4$ and concentrated under reduced pressure. The resultant solid obtained was triturated with 150 mL of diethyl ether, filtered, and washed with diethyl ether to obtain pure PBHP monomer as a yellow solid 1.3gm (5.3mmol, 89% yield). Spectral data was identical to previous report.[1] ^{1}H NMR (400 MHz, $CDCl_3$) δ 8.45 (d, *J*= 4.0 Hz, 2H), 8.01 (s, 4H), 6.29 (d, *J* = 4.0 Hz, 2H).^{13}C NMR (100 MHz, $CDCl_3$) δ 185.64, 180.05, 138.50, 127.74, 98.93

<u>Synthesis of of 1,3,5-tris(4-aminophenyl)triazine (4) (TAPT):</u>

1,3,5-tris-(4-aminophenyl)triazine was synthesized *via* trimerization catalyzed by trifluromethanesulphonic acid. In a typical synthesis, 2.0 g (16.94 mmol) 4-aminobenzonitrile was taken in a round bottom flask at 0 ºC. Then 4.48 mL (50.82 mmol) trifluoromethanesulfonic acid was added dropwise at 0 ºC. The resultant mixture was stirred for 24 h at room temperature in inert atmosphere. After completion the reaction mixture was neutralized using 2 N NaOH, The resultant white solid obtained was filtered and washed several times with water to obtain pure TAPT monomer as white solid 1.64 gm (4.6 mmol, 89% yield). The spectral data obtained was identical to previous reports.[2] ^{1}H NMR (400 MHz, DMSO-d_6) δ 8.36 (d, *J* = 8.4 Hz, 6H), 6.70 (d, *J* = 8.4 Hz, 6H), 5.90 (s, 6H). ^{13}C NMR (100 MHz, DMSO-d_6) δ 170.03, 153.43, 130.60, 123.39, 113.57.

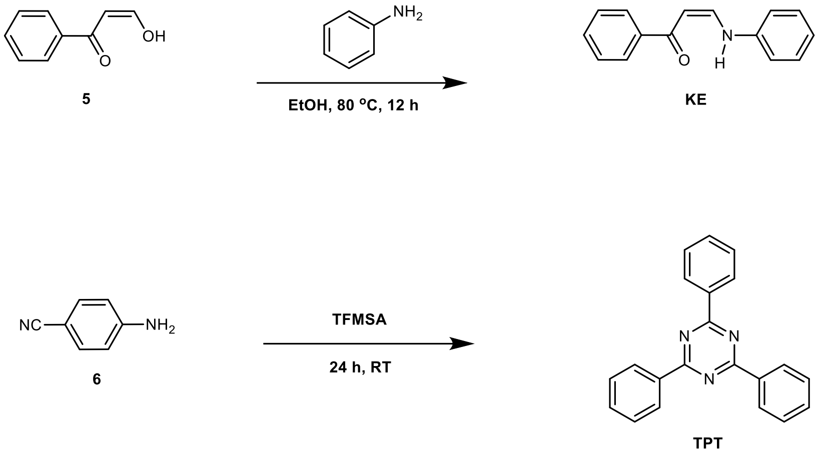

Supplementary Figure 2. Synthesis of model compounds keto-enamine (KE) and triphenyltriazine (TPT)

<u>Synthesis of (*Z*)-3-hydroxy-1-phenylprop-2-en-1-one (5) (mono-keto-enol):</u>

A solution of Acetophenone (1 g, 8.33 mmol) in 80 ml of anhydrous THF was added drop-wise to tBuOK (2.33 g, 20.82 mmol in 100 ml of anhydrous THF) at -78 ^{o}C for 1hr under Argon atmosphere. After stirring at the same temperature for additional 1 hr a light yellow color solution was obtained. To this ethyl formate (3.08 g, 4.95 mL, 41.52 mmol) was added and allowed the reaction mixture to warm up to room temperature and continued the stirring for 12 hrs. The reaction was quenched with 3N HCl (60 ml) and the organic layer were extracted with ethyl acetate (2x100 mL) dried over $MgSO_4$ and concentrated under reduced pressure. The resultant dark brown solid was found to be unstable and used in the next step without further purification. Structure confirmed by ^{1}H NMR, the spectral data was identical to previous reports.[1] ^{1}H NMR (400 MHz, $CDCl_3$) δ 8.31 (d, J = 4.2 Hz, 1H), 7.93 (dd, 2H), 7.60 – 7.54 (m, 1H), 7.52 – 7.45 (m, 2H), 6.24 (d, J = 4.2 Hz, 1H).

<u>Synthesis of (*Z*)-1-phenyl-3-(phenylamino)prop-2-en-1-one (KE):</u>

A solution of Aniline (0.1 g, 1.07 mmol) dissolved in 2 ml of anhydrous ethanol was added drop-wise to a solution of (Z)-3-hydroxy-1-phenylprop-2-en-1-one (mono-keto-enol , 0.159 g, 1.07 mmol) in 20ml ethanol and 6N AcOH (0.2ml) at room temperature. After stirring for 10 min, the reaction mixture was refluxed at 80 ^{o}C for 12 h under Argon atmosphere. After completion, the reaction mixture was cooled to room temperature and the solid product was filtered off and washed with ethanol several times, to obtain pure keto-enamine (KE) as a yellow solid 0.215 gm (0.9 mmol, 90% yield). The spectral data obtained was identical to previous reports.[3] ^{1}H NMR (400 MHz, $CDCl_3$) δ 12.15 (s, 1H), 7.96 (d, J = 6.8 Hz, 2H), 7.61 – 7.43 (m, 5H), 7.42 – 7.31 (m, 2H), 7.19 – 7.04 (m, 3H), 6.06 (d, J = 7.8 Hz, 1H).

^{13}C NMR (100 MHz, $CDCl_3$) δ 191.02, 144.96, 140.24, 139.19, 131.59, 129.76, 128.45, 127.32, 123.72, 116.36, 93.72

Synthesis of 2,4,6-triphenyl-1,3,5-triazine (TPT):

In a typical synthesis 1.0 g (9.6 mmol) benzonitrile was taken in a round bottom flask at 0ºC. Then 2.53 mL (28.8 mmol) trifluoromethanesulfonic acid was added dropwise at 0 ºC. The resultant mixture was stirred for 24 h at room temperature in inert atmosphere. After completion the reaction mixture was neutralized using 2M NaOH, The resultant white solid obtained was filtered and washed several times with water to obtain pure TPT monomer as white solid 0.82gm, (2.6mmol, 82% yield). The spectral data obtained was identical to previous reports.[4]

^{1}H NMR (400 MHz, $CDCl_3$) δ (ppm): 8.84 – 8.80 (m, 6H), 7.67-7.58 (m, 9H).

^{13}C NMR (100 MHz, $CDCl_3$) δ (ppm): 171.66, 136.25, 132.52, 128.97, 128.65.

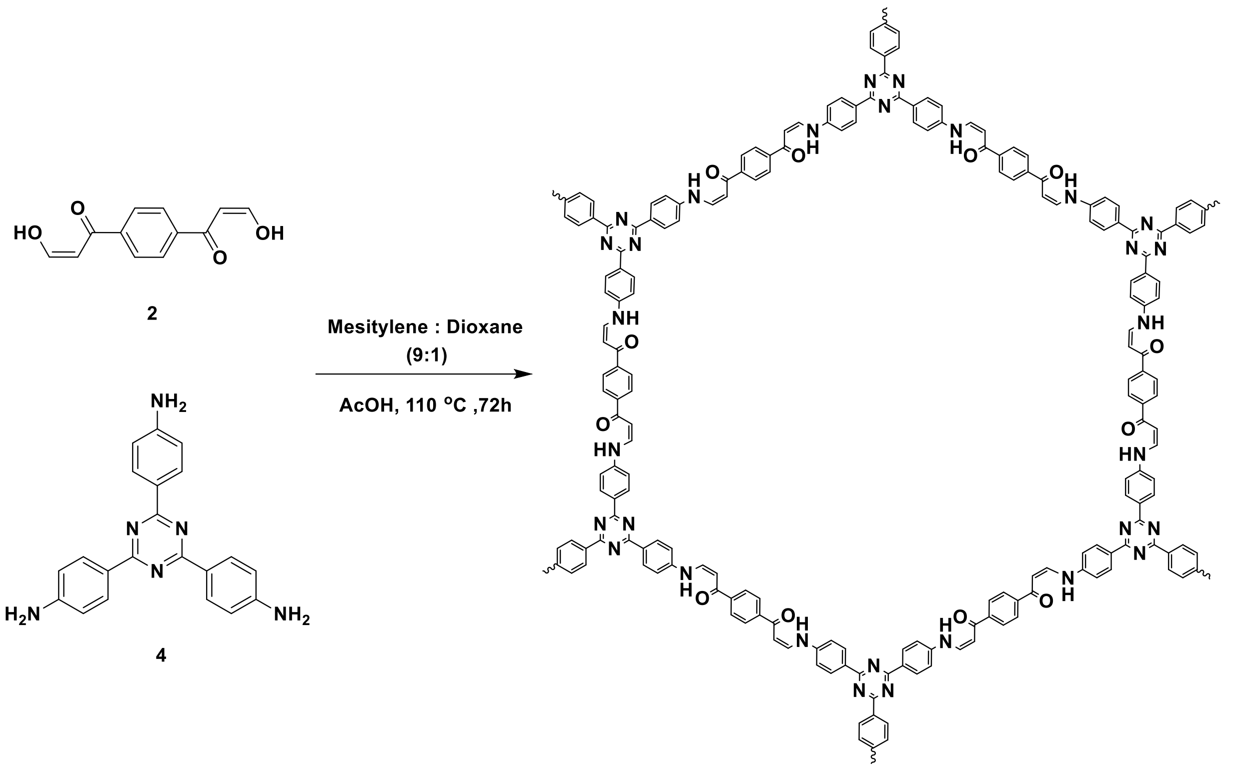

Supplementary Figure 3. Synthesis of PBHP-TAPT COF

Triazine containing 2D covalent organic frameworks was synthesized according to method described by Perepichka et al.[1] All the trails for synthesis of COFs were carried out in a Pyrex ampule of OD: 2.6 mm, ID: 2.4 mm. In a typical synthesis mixture of TAPT (71 mg, 0.2 mmol) and PBHP (65 mg, 0.3 mmol) and 6 M aq. acetic acid (0.5 mL) were suspended in a degassed mixture of Mesitylene and Dioxane. The mixture was sonicated for 10 min, flash frozen in liquid N_2 and degassed for 10 min. The ampule was then sealed and heated to 110 °C for 3 days. After the reaction the ampule was broken and the contents were filtered and washed thoroughly with ethanol, dioxane and methanol to get rid of any oligomer impurities as well as unreacted monomers. Furthermore, the solid was then subjected to soxhlet extraction using methanol for 72 h, which gave an orange powder PBHP-TAPT COF 98 mg, (0.0780mmol calculated for unit cell $C_{(78)}H_{(54)}N_{(12)}O_{(6)}$, 79.9% yield). The isolated solid PBHP-TAPT COF was insoluble in water and common organic solvents such as acetone, ethanol, and *N*,*N*-dimethylformamide.

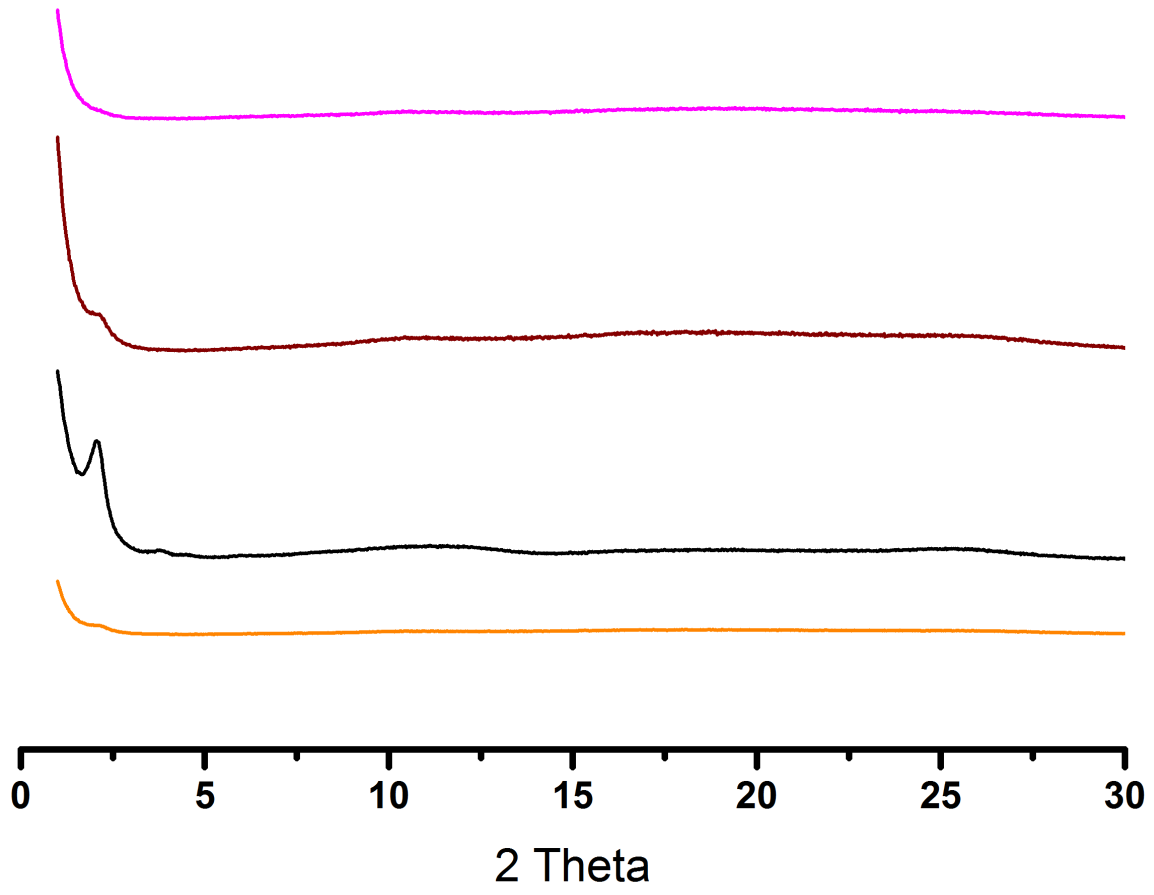

Supplementary Figure 4. Comparison of the observed PXRD patterns of PBHP-TAPT COF with different ratios of dioxane/mesitylene (v/v). Mesitylene only (pink), 19:1 (maroon), 9:1 (black), and 5:1 (orange). The optimized ratio of dioxane/mesitylene (v/v) is 9:1, which results in the material with highest crystallinity (black).

Supplementary Table 1. Combustion elemental analysis (EA) data for PBHP-TAPT COF

sample		C (%)	H (%)	N (%)	O (%)
PBHP-TAPT COF	Theoretical	74.63	4.34	13.39	7.65
	Experimental	71.36	4.47	12.56	ND

*ND is not determined

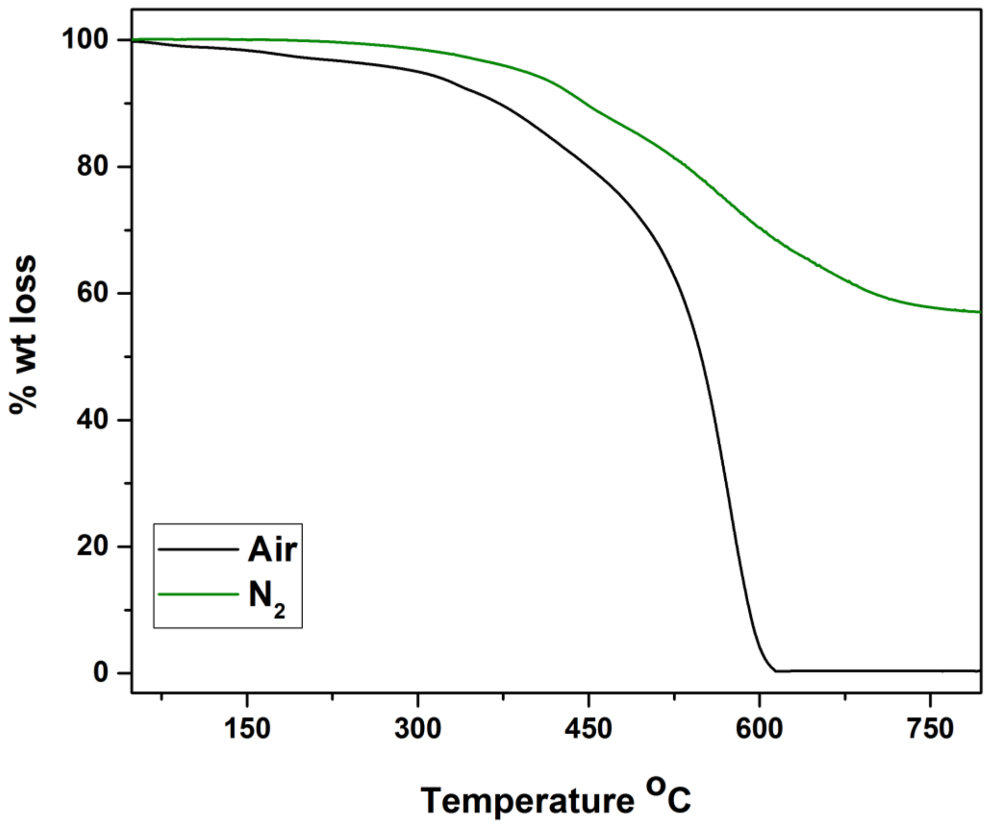

Supplementary Figure 5. Thermogravimetric analysis of PBHP-TAPT COF under air and nitrogen, observed residual mass was 2% and 40% at 800 ^{o}C under air and nitrogen respectively.

a)

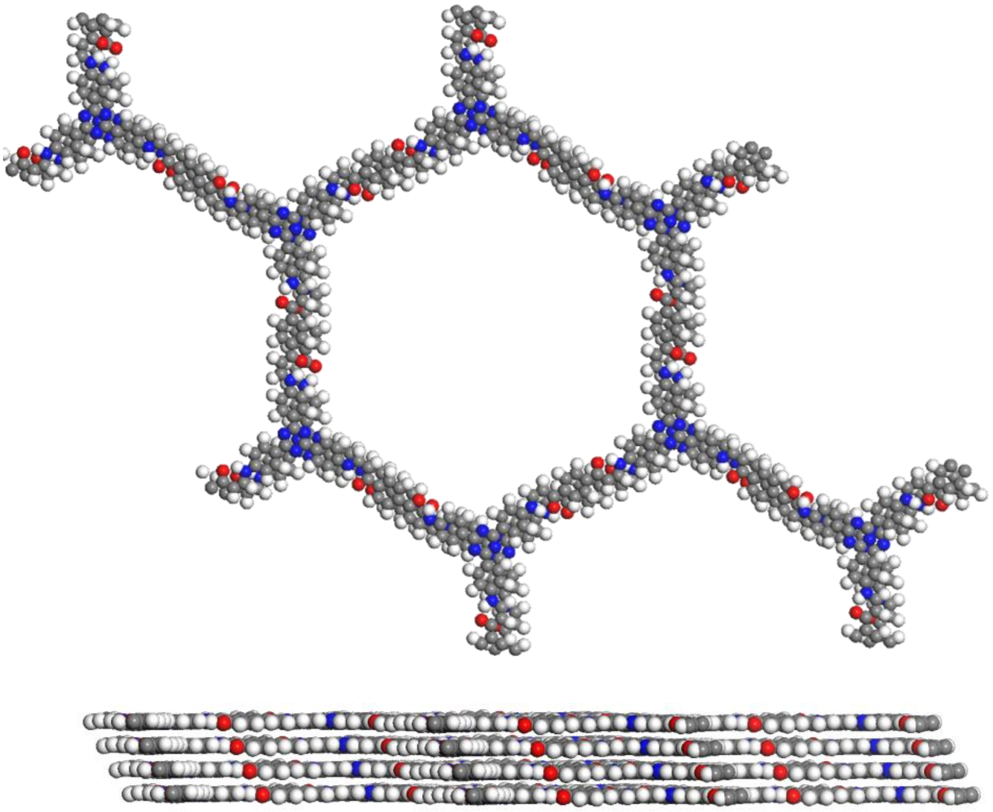

b)

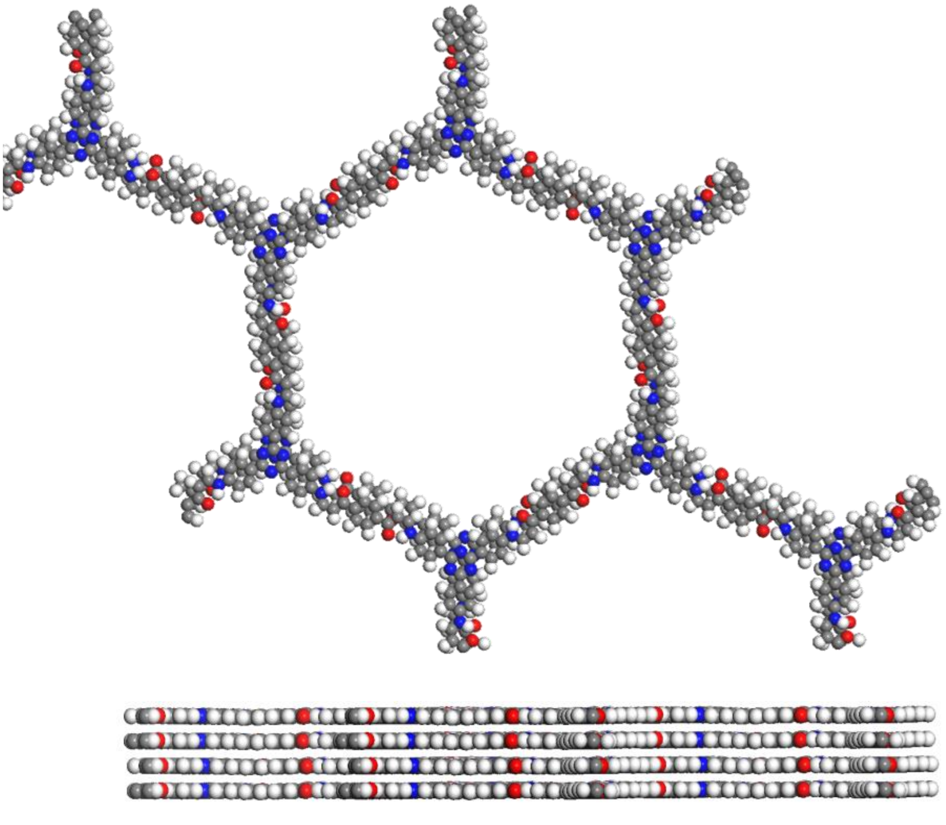

c)

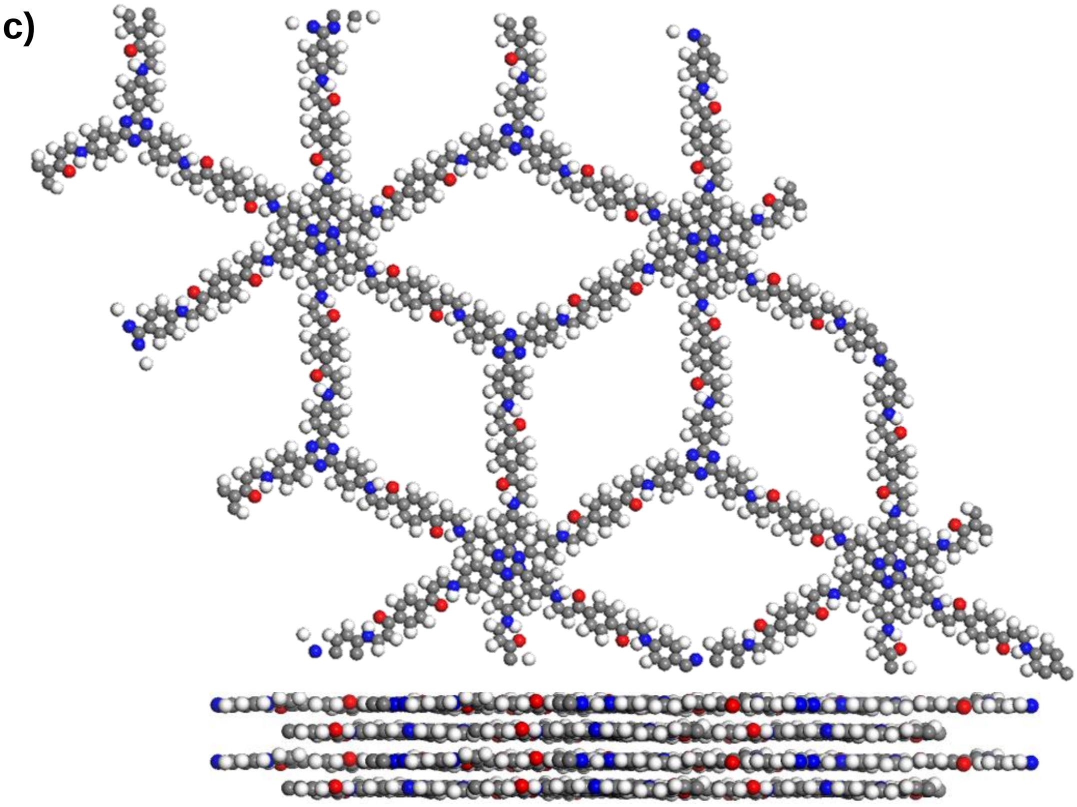

Supplementary Figure 6. DFT model structures used for PXRD assignment a) AA-serrated, b) AA-eclipsed and c) AB-staggered.

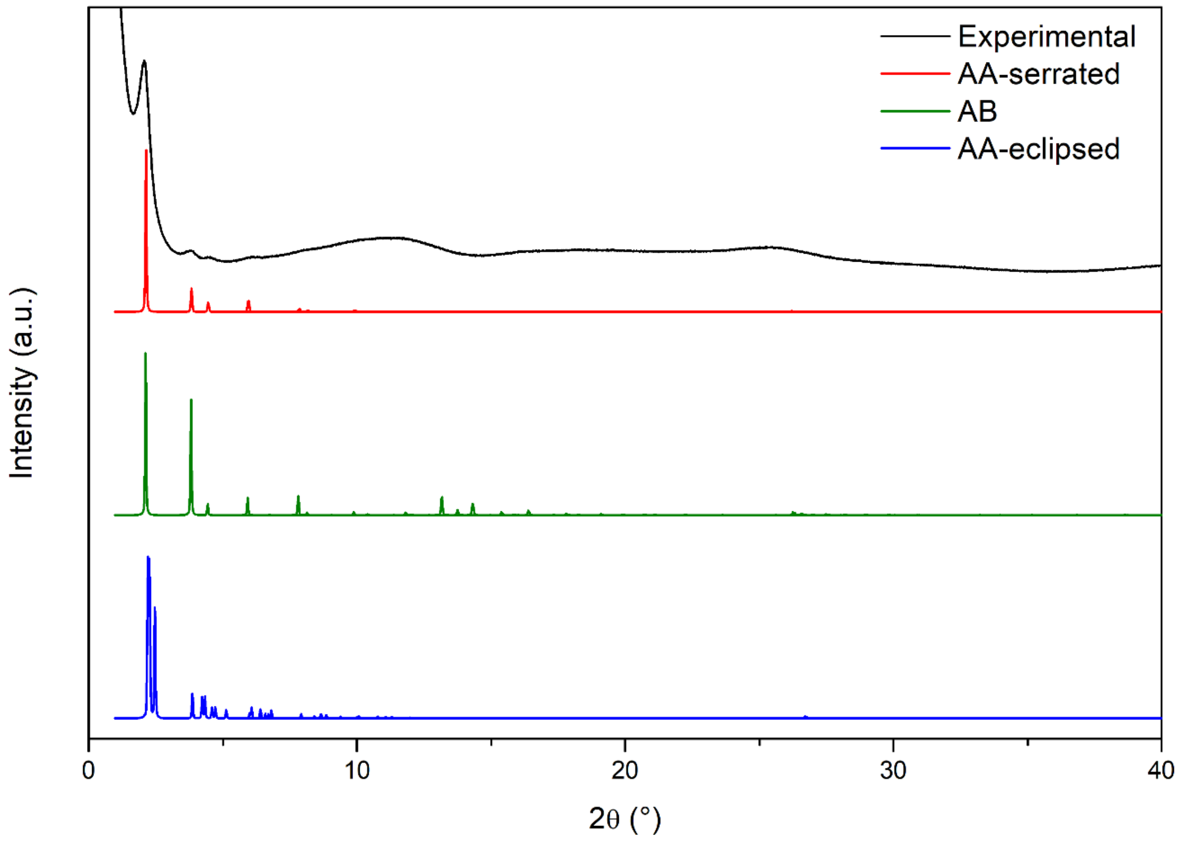

Supplementary Figure 7. Comparison of PXRD pattern of PBHP-TAPT COF (black) and corresponding simulated patterns for AA-Eclipsed, AA-Serrated and AB-Staggered (accounting for zero-point instrumental shift obtained from Pawley refinement).

Supplementary Table 2. The relative energy E_{rel} (per UC), lattice constants (a, b, c, α, β and γ) and interlayer distance calculated for AA-eclipsed, AA-serrated and AB structures (see Supplementary figure 6 and 7 for definition).[a]

	E_{rel}	Lattice constant						Interlayer distance
		a	b	c	α	β	γ	
AA-eclipsed	0	44.42	44.05	7.65	63	115	120	3.32
AA-serrated	+0.10	44.35	43.96	6.76	88	94	120	3.37
AB	+4.30	44.63	44.06	5.98	89	91	120	2.99

[a] Energies, distances and angles are reported in eV, Å and deg

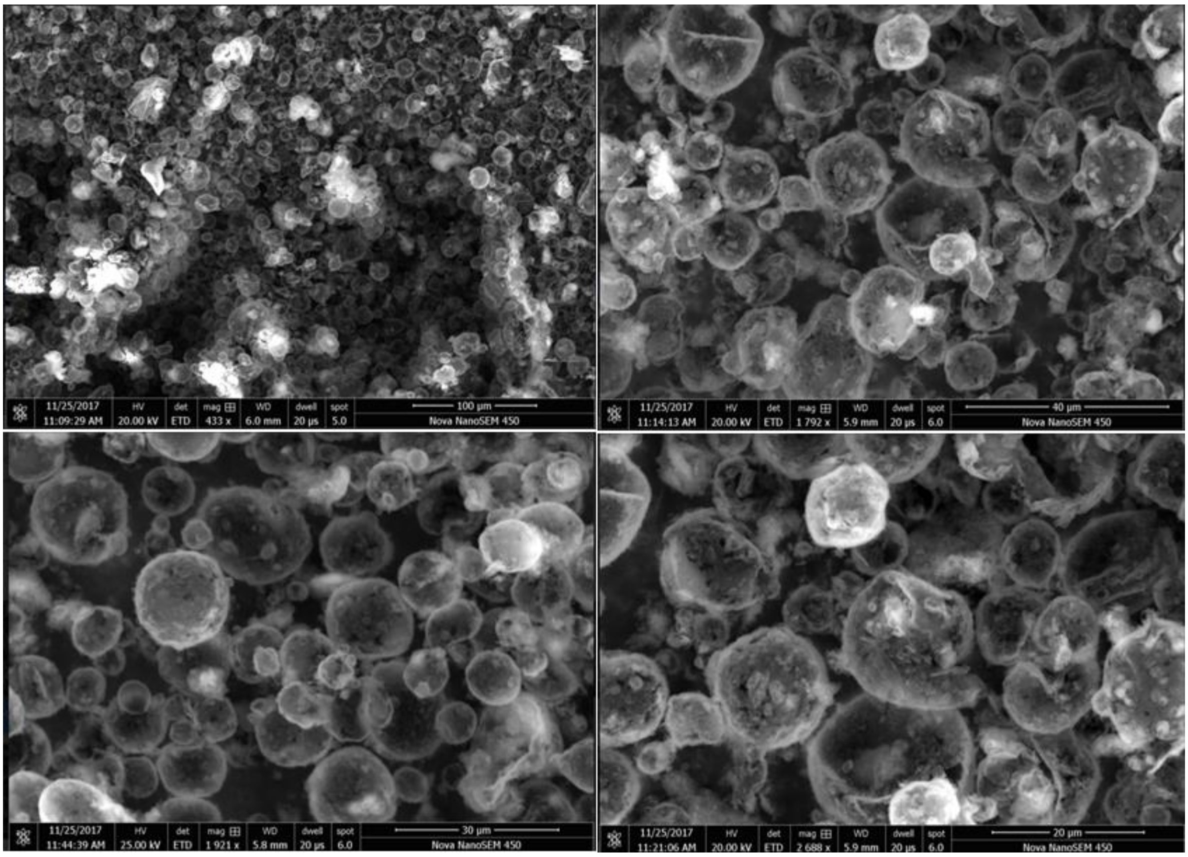

Supplementary Figure 8. SEM images of PBHP-TAPT COF powders after purification and drying collected at an accelerating voltage of 20 kV.

a)

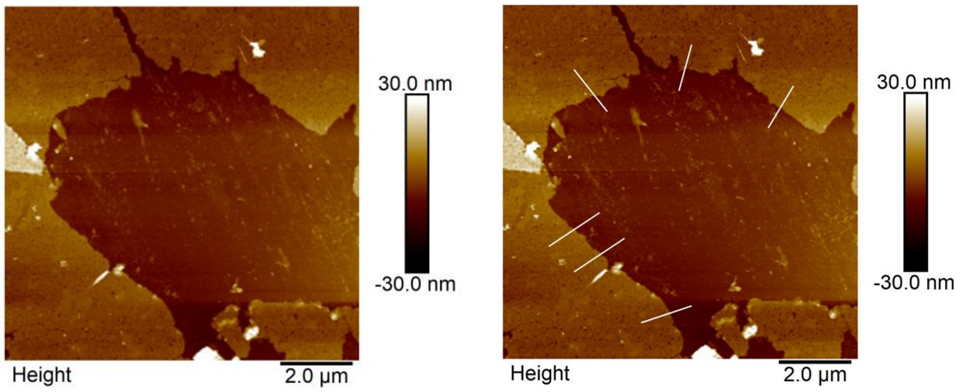

b)

Spot	Height
1	11.7
2	9.8
3	11.5
4	8.3
5	10.6
6	7.03
Average	9.8
STD	1.8

c)

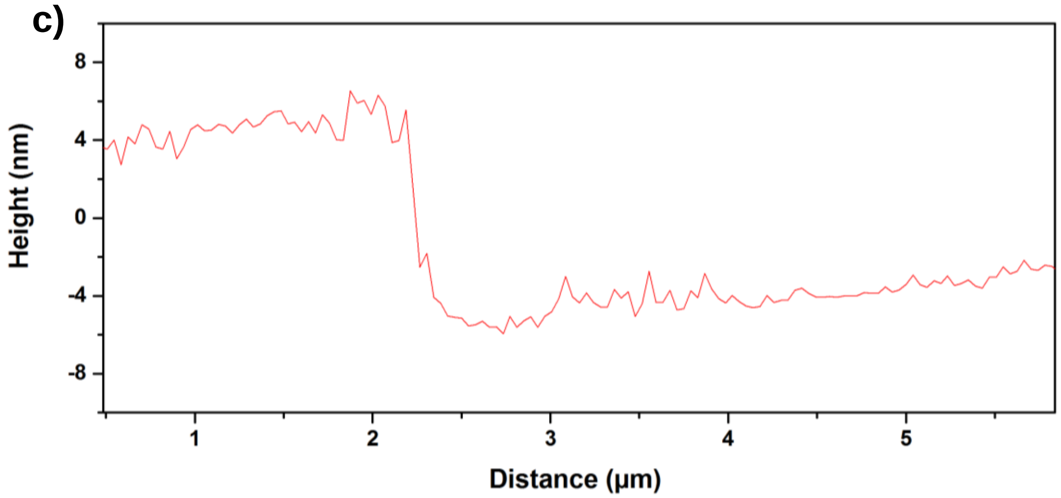

Supplementary Figure 9. a) AFM images, b) height analysis and c) a typical height profile of exfoliated PBHP-TAPT COF

The PBHP-TAPT COF powder was suspended in THF and sonicated for 30 min in an ultrasonicator. Then the supernatant solution was drop casted onto a silicon substrate (with 300 nm-SiO_2 layer). Peak Force Tapping mode was employed with the cantilever SCANASYST-AIR.

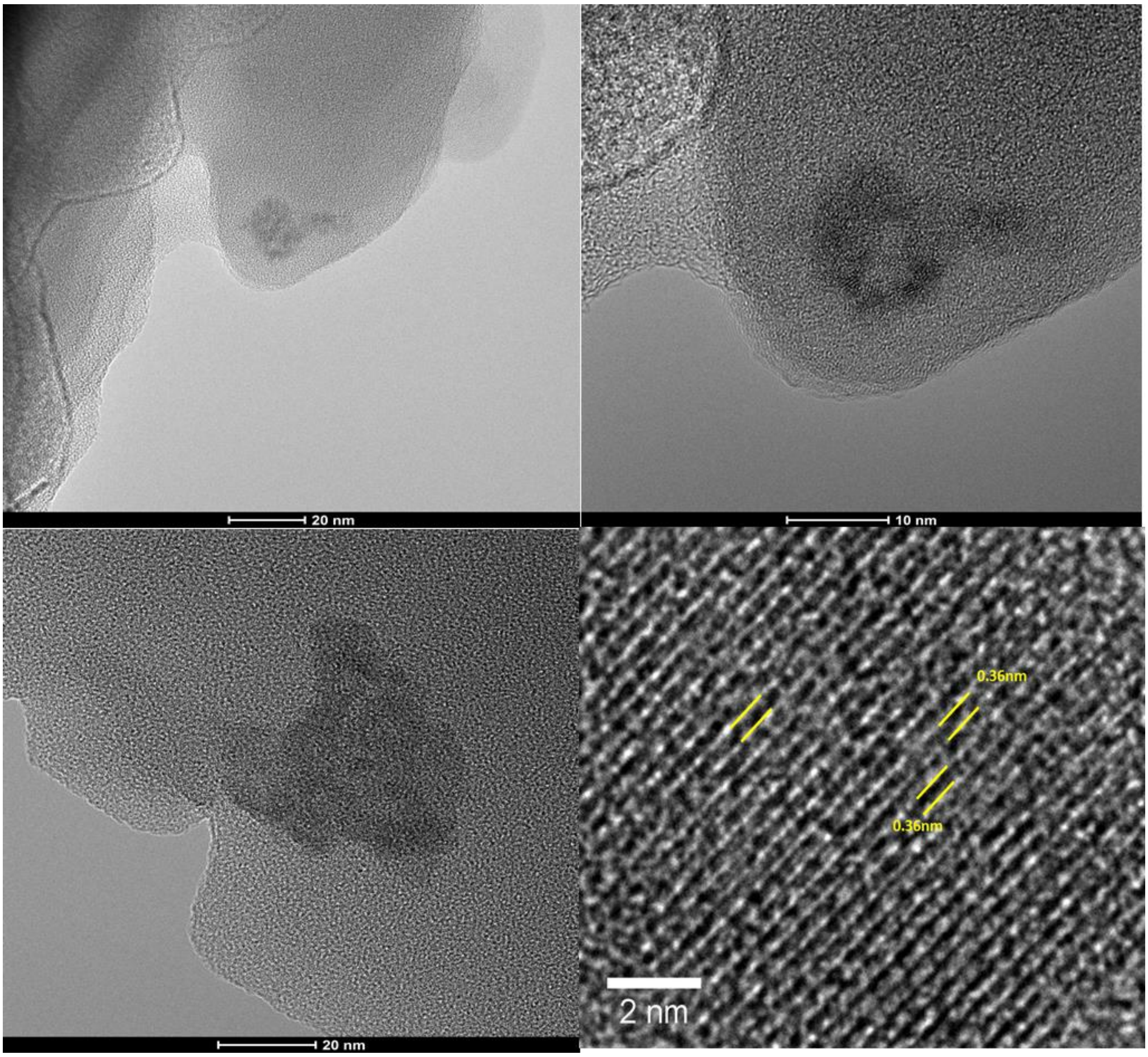

Supplementary Figure 10. TEM images of PBHP-TAPT COF powders after purification and drying collected at an accelerating voltage of 200 kV.

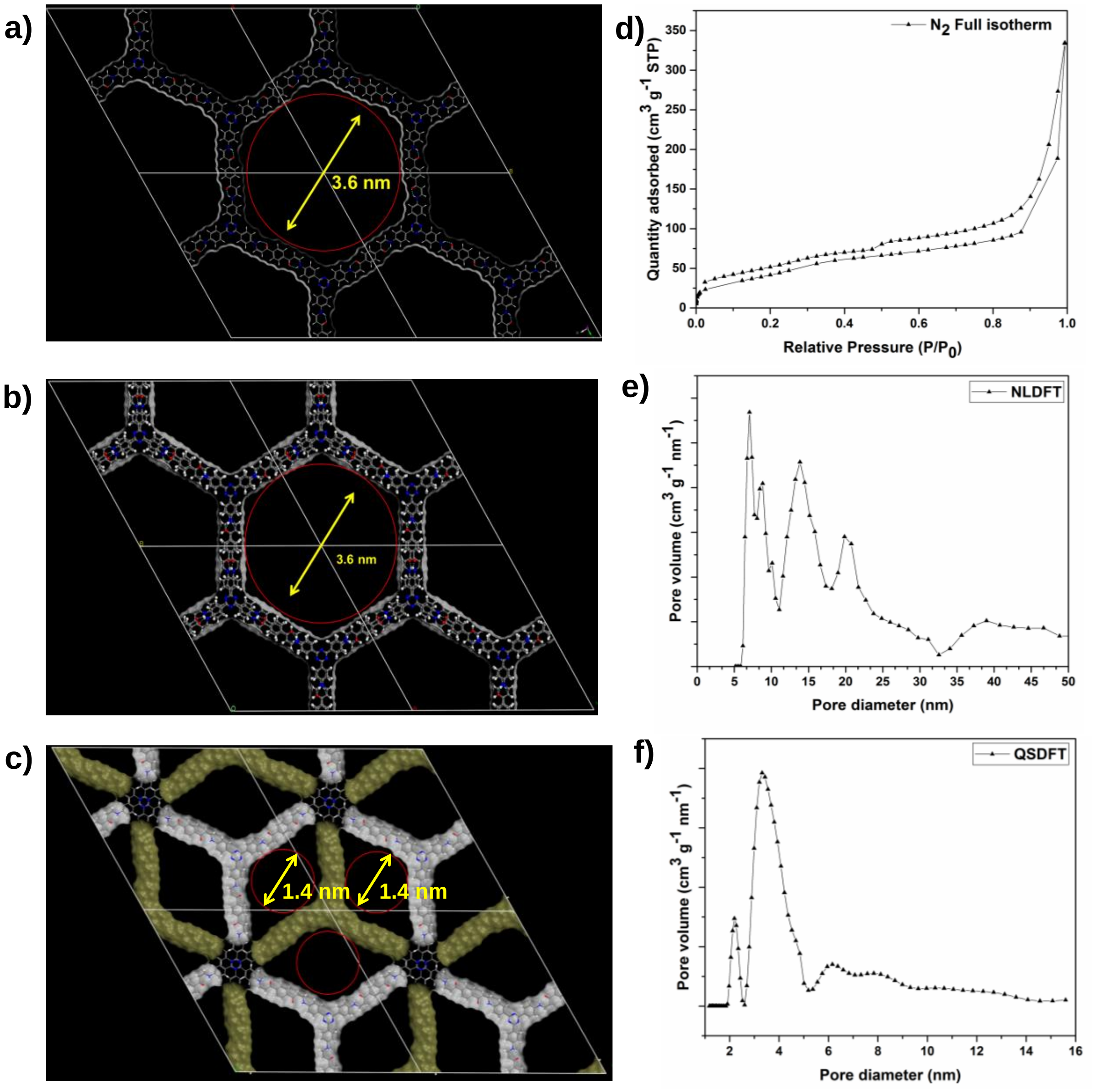

Supplementary Figure 11. Calculated pore diameter using Connolly surface function for a)AA-Eclipsed, b) AA'-serrated, c) AB-staggered stacking modes of PBHP-TAPT COF, d) Nitrogen adsorption isotherm, e) pore width analysis using NL-DFT and f) pore width analysis using QS-DFT models.

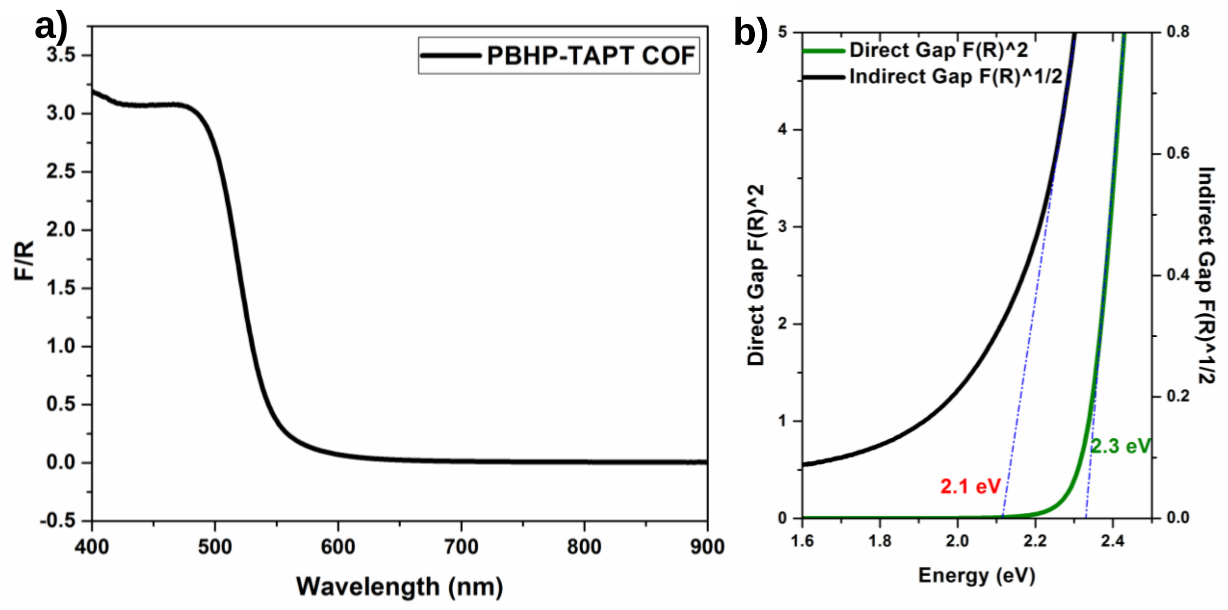

Supplementary Figure 12. a) Solid-state UV-Vis of PBHP-TAPT COF and b) Kubelka-Munk plots for PBHP-TAPT COF assuming direct (dark) and indirect (light) band gap.

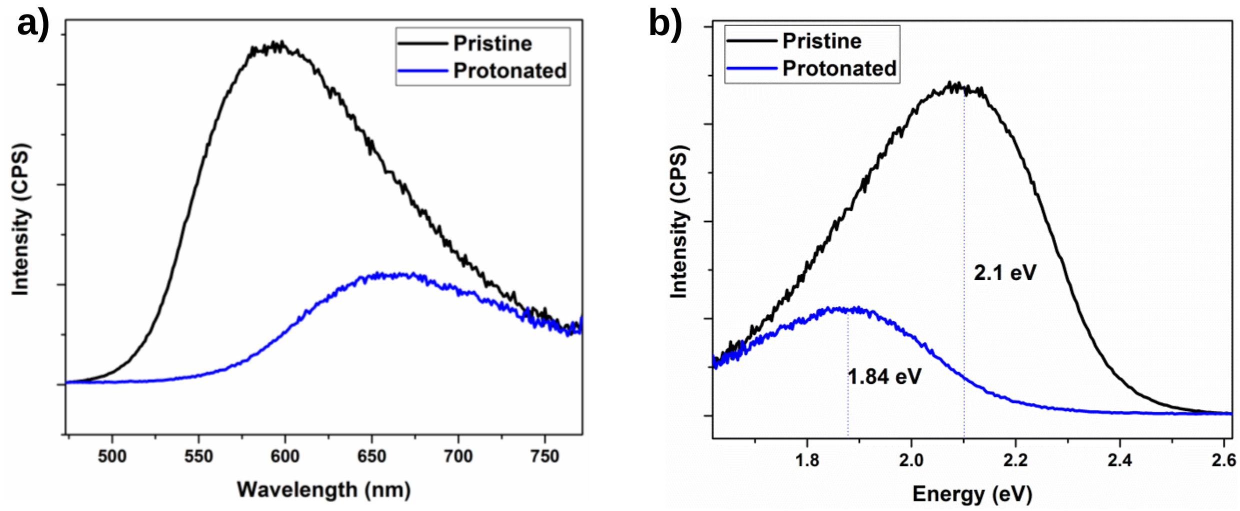

Supplementary Figure 13. a) Solid-state photoluminescence emission spectra of pristine and protonated PBHP-TAPT COF (Exposed to HCl gas for 10 sec) and b) correlation of observed PL transition with band gap (eV).

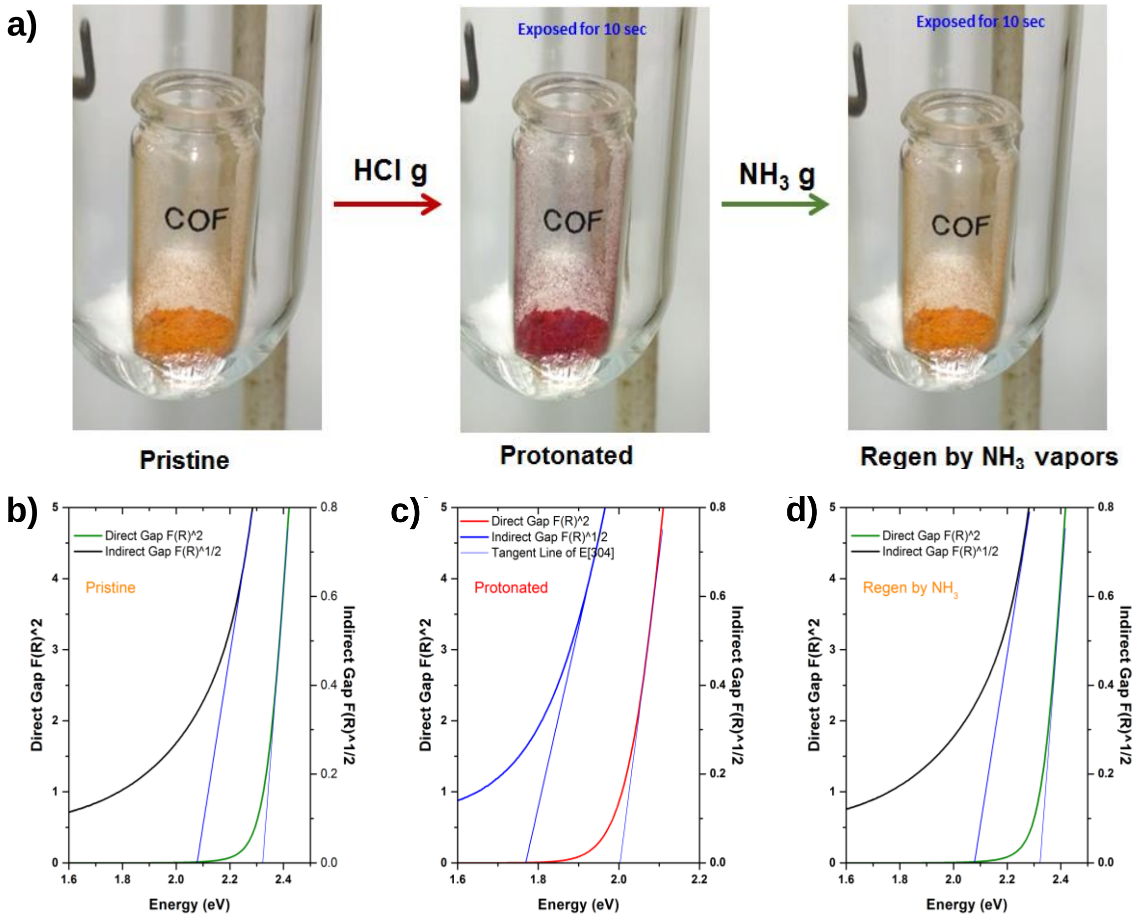

Supplementary Figure 14. a) Photograph of sequential color change of PBHP-TAPT COF on exposures of HCl and NH_3 vapors PBHP-TAPT COF showed a response to HCl (g) within 7 s, and Kubelka Munk plots assuming direct and indirect optical band gaps of PBHP-TAPT COF before and after gas phase protonation/deprotonation of b) pristine, c) protonated by HCl vapors and d) followed by regeneration with NH_3 (Supplementary Movie 1).

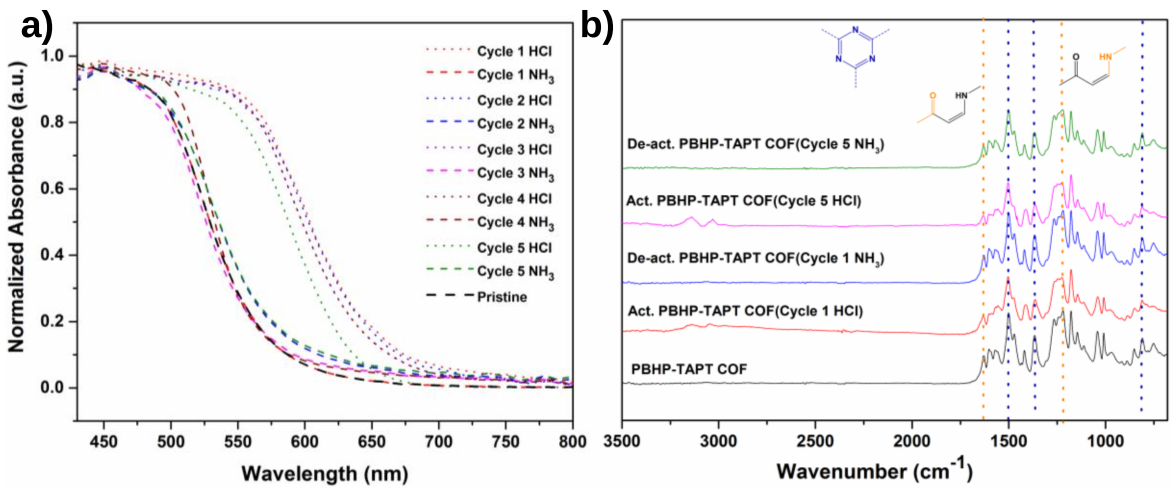

Supplementary Figure 15. a)UV-Vis diffusive reflectance spectra of cycling of PBHP-TAPT COF, by HCl vapour and regeneration using NH_3 vapors and b) FT-IR spectra after cycling.

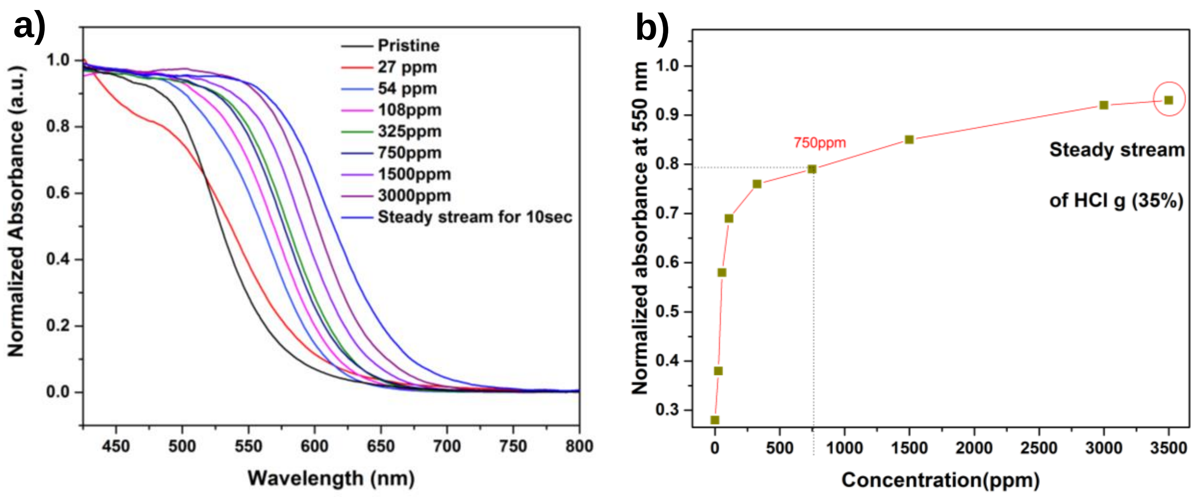

Supplementary Figure 16. a) HCl vapour concentration study of PBHP-TAPT COF solid-state UV-Vis spectra COF, by hydrochloric acid vapour at different concentrations within 10 s at 25 ^{o}C and b) Absorbance vs concentration curve at 550 nm.

Supplementary Note 1.

Colorimetric detection limit of PBHP-TAPT COF towards HCl vapors was carried out using a glass solution bottle with a stopper COF was exposed to vapours of hydrochloric acid in temperature controlled environment by keeping the reaction vial in the oil bath (25 ^{o}C monitored with a thermometer). Smaller vial with sample was introduced inside the reaction vial containing necessary amount of HCl. Samples were exposed for 10 s and further subjected to solid-state UV-Vis measurements without any delays. HCl vapour concentration in ppm was calculated by following equation:

Supplementary Equation 1.

$$ppm\ (HCl) = \frac{\mu g\ (HCl\ vapor)}{g\ (air)} \tag{1}$$

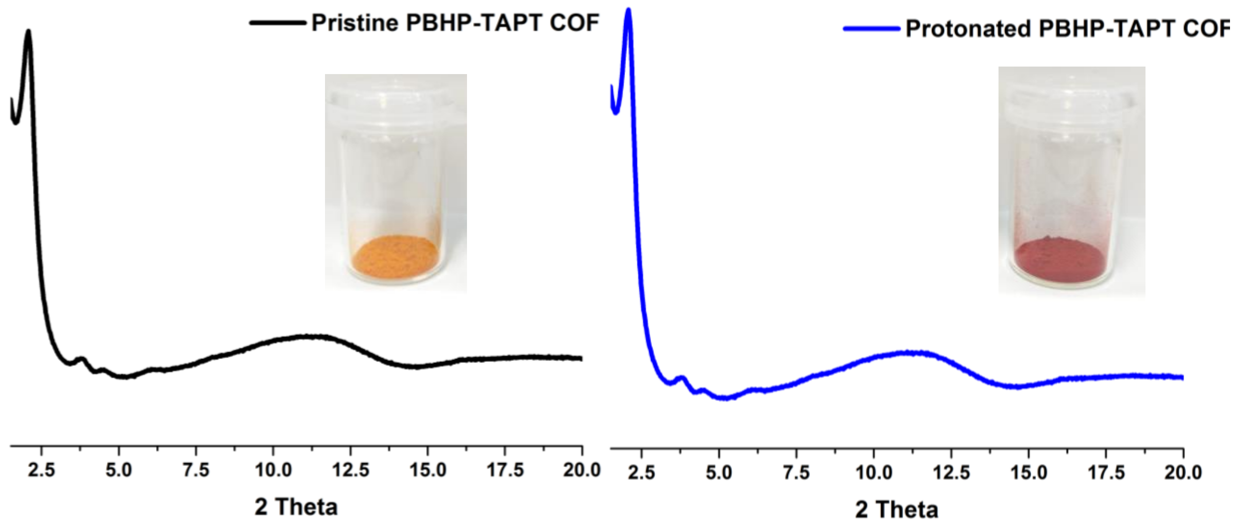

Supplementary Figure 17. PXRD patterns of Pristine and protonated PBHP-TAPT COF (exposed to steady stream of HCl gas for 30 s) and inset photograph of respective PBHP-TAPT COF samples.

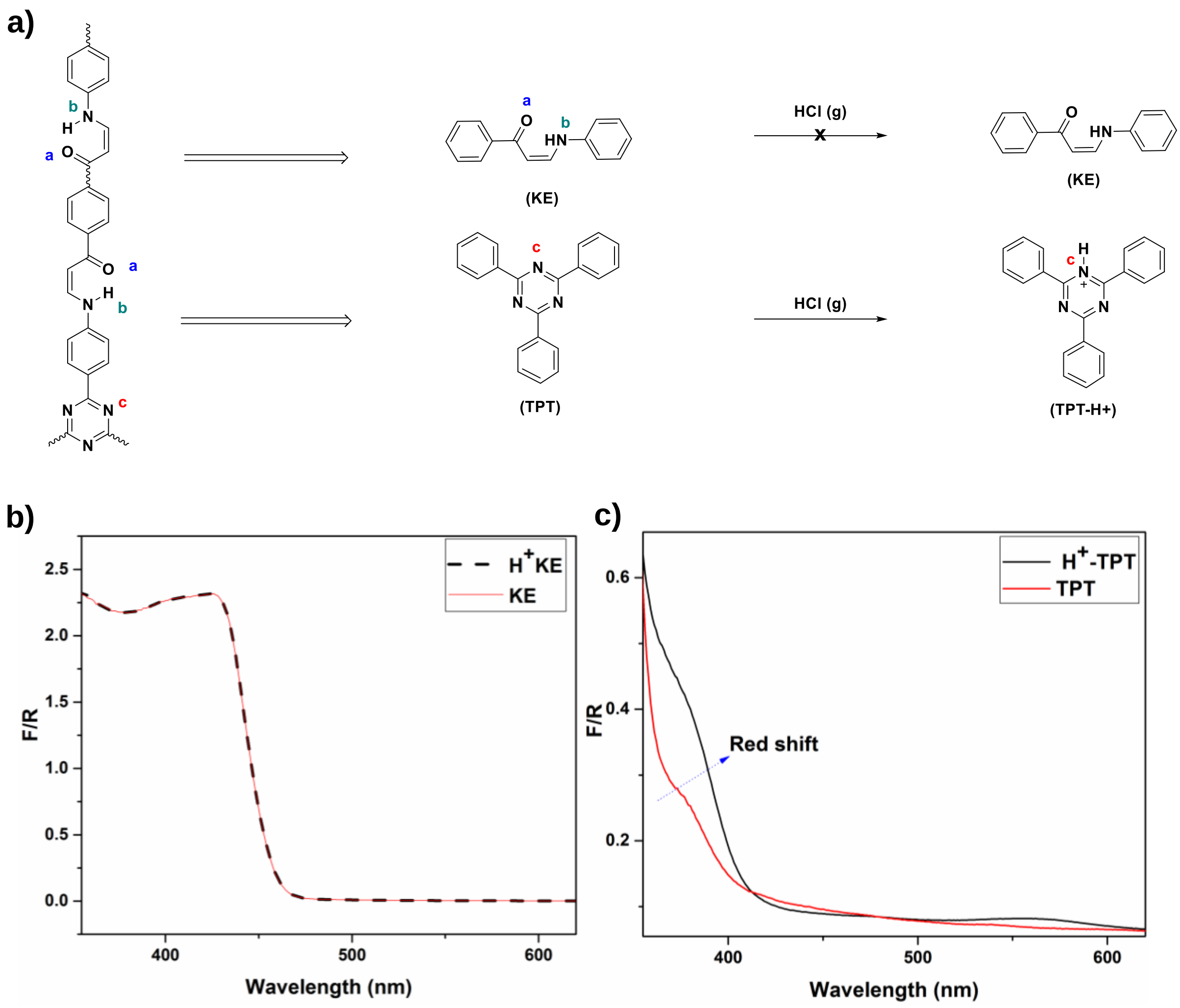

Supplementary Figure 18. a) Sub-units of PBHP-TAPT COF with conceivable protonation sites (a, b, c), b) solid-state UV/Vis spectrum of of keto-enamine (KE) model compound before (in red) and after exposure to HCl (g) (in black, dashed) d) solid-state UV/Vis spectrum of triphenyl triazine (TPT) model compound before (in black) and after (in red) exposure to HCl (g) treatment.

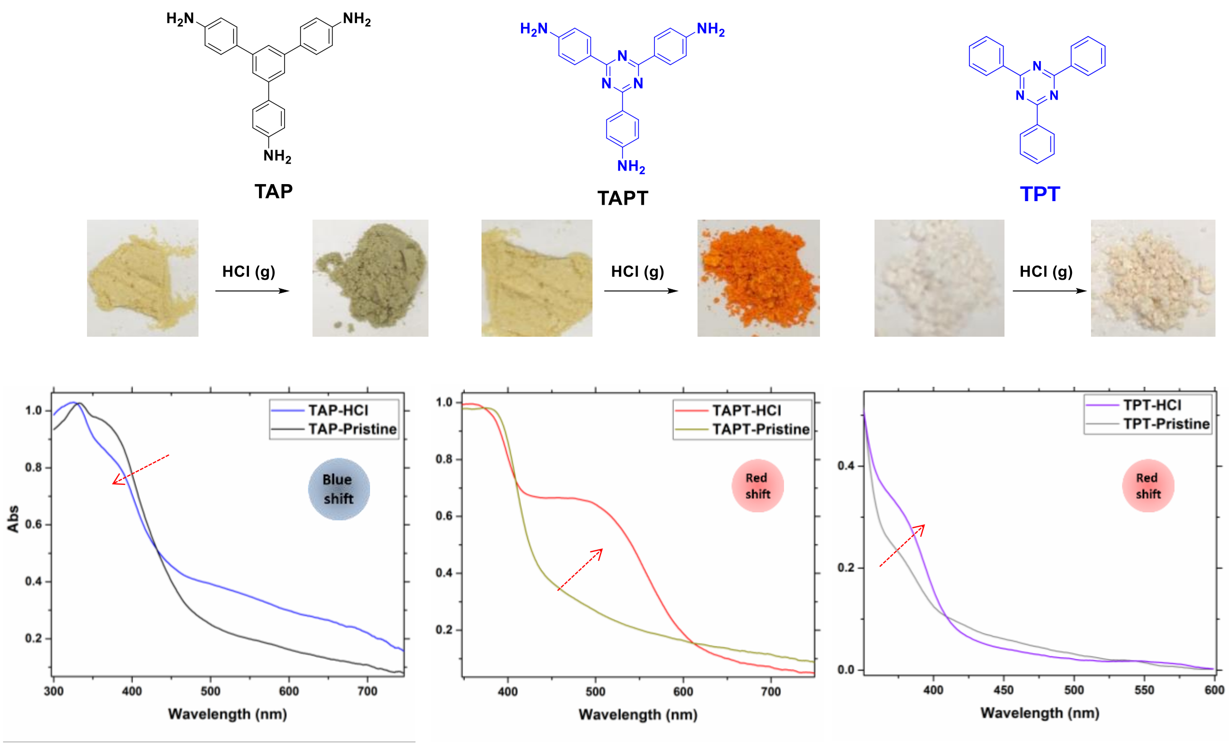

Supplementary Figure 19. Solid-state UV/Vis measurements of the model compounds: 1,3,5-tris(4-aminophenyl)benzene (TAP-Amine); 1,3,5-tris-(4-aminophenyl) triazine (TAPT-Amine); and 2,4,6-triphenyl-1,3,5-triazine (TPT). Protonation was performed using a stream of HCl (g) for 10 s.

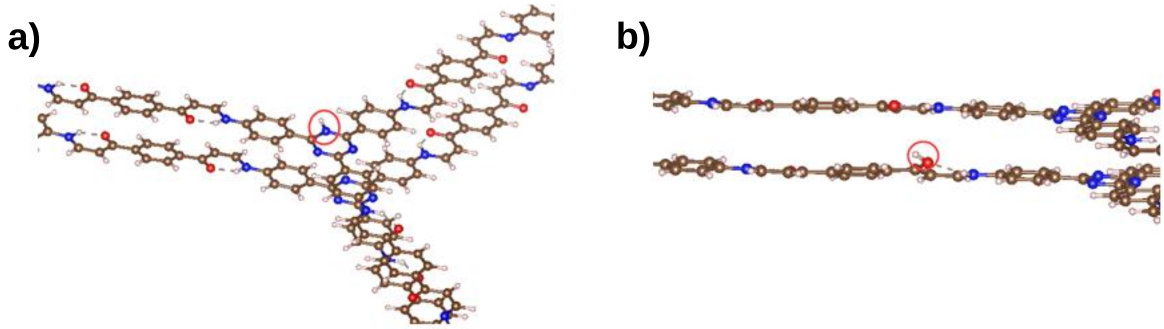

Supplementary Figure 20. The protonation of (a) the triazine and (b) the bridge of the PBHP-TAPT COF. Position of added proton is shown within the red circle. Color scheme: H (white), C (brown), N (blue) and O (red)

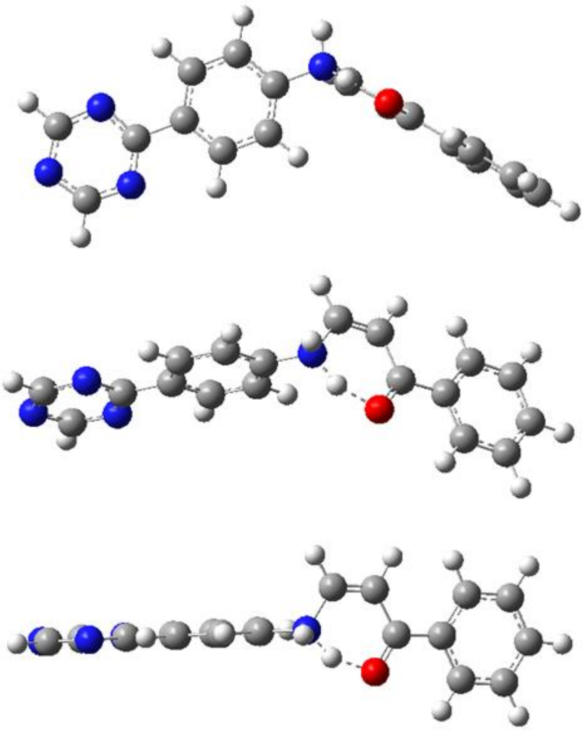

Supplementary Figure 21. The cluster model for protonation of the linker; view from different directions; grey, blue, red and white balls represent C, N, O and H, respectively.

Supplementary Note 2.

The protonation of PBHP-TAPT COF has been investigated computationally (Supplementary Figure **20**), using the same models and methods as described above in computational details in the main text. Protonation sites on the N atom of triazine and on the N atom of the keto-enamine linker were considered. The DFT results show that the protonation on triazine is preferred by 70 kJ mol^{-1} over protonation on the keto-enamine linker. The proton added on the N atom of triazine is not involved in any H-bonding. On the contrary, the proton added to the N atom of the keto-enamine linker is stabilized by the formation of the H-bond with the O atom of the linker at the adjacent layer. At the minimum energy structure, the proton is shifted from N to O atom of the keto-enamine linker. We carried out the same calculations using cluster model (Supplementary Figure **21**) consisting of triazine-linker. While the relative energies of protonated connector and linker was similar (77 kJ mol^{-1} in favor of triazine protonation), the minimum energy structure was different: H is primarily bound to the N atom and it forms an H-bond with the O atom which is moved into the optimal position for H-bonding (a planar O-C-C-C-N-H ring. Such rearrangement requires rotation along two backbone C-N bonds which is forbidden in the 2D PBHP-TAPT COF (adjacent connectors would not be in the same plane). Thus, the rigidity of 2D PBHP-TAPT COF leads to the proton transfer from N to O atom of the linker.

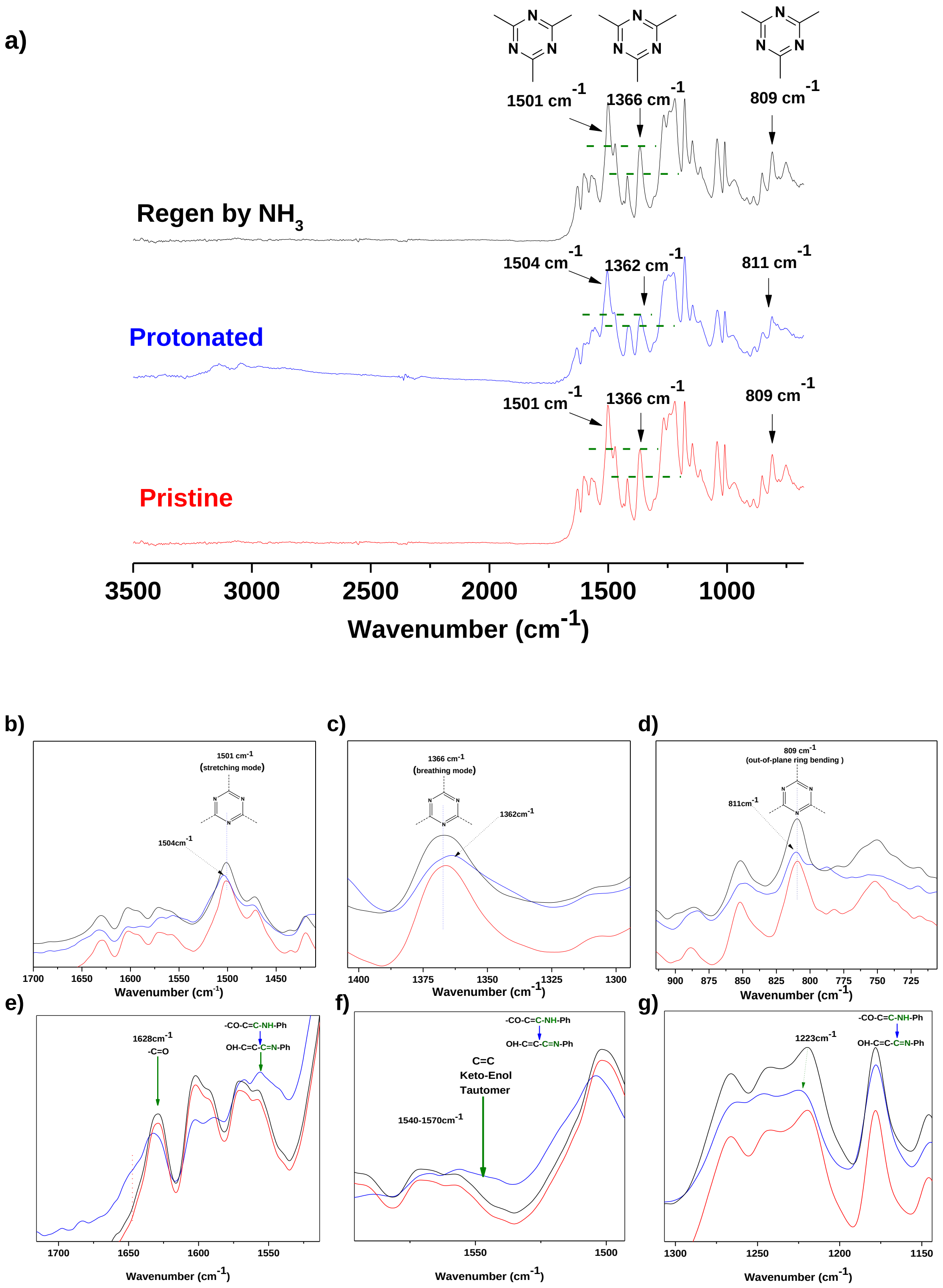

Supplementary Figure 22. Fourier-transform infrared (FT-IR) spectroscopic study on protonation and deprotonation of PBHP-TAPT COF, pristine PBHP-TAPT COF, protonated PBHP-COF (activation with HCl vapours) and regeneration by NH_3 for the conformation of protonation site and characteristics of triazine and keto-enamine regions affected due to protonation by HCl gas.

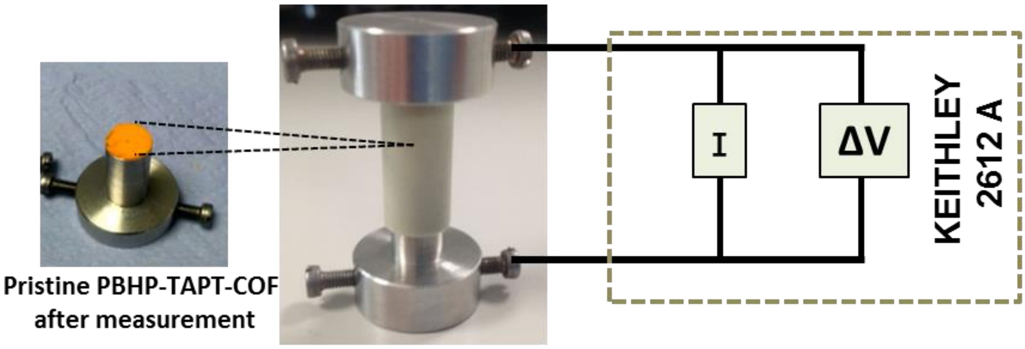

Supplementary Figure 23. Photograph of conductivity measurement kit and pressed pellet after measurement

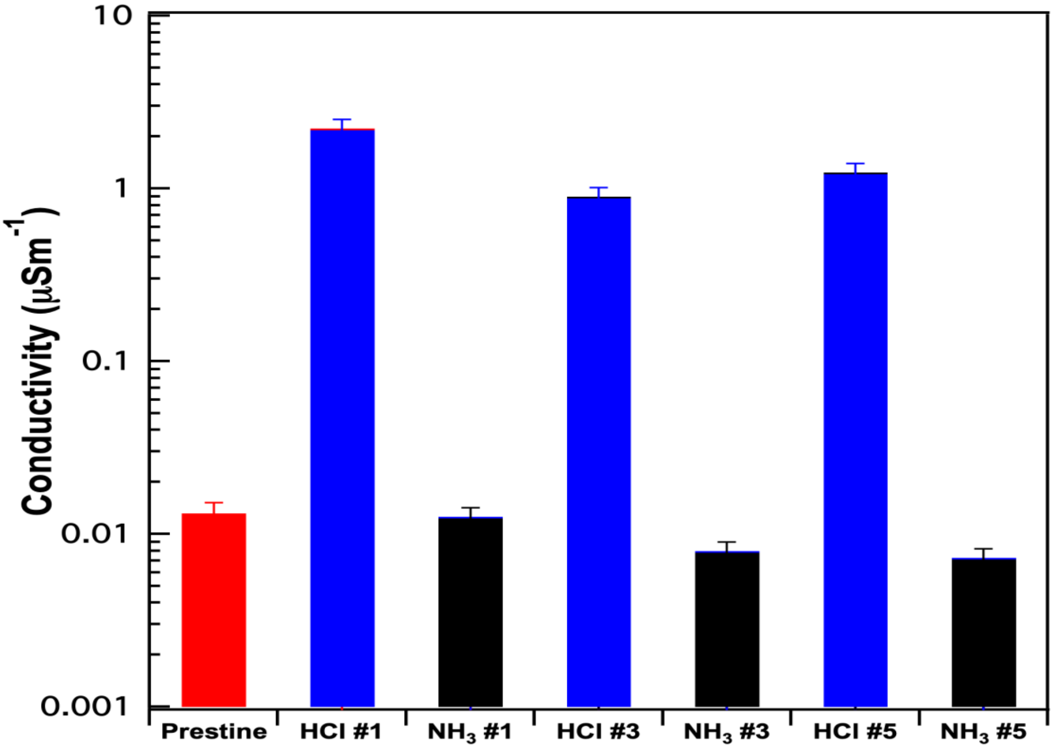

Supplementary Figure 24. Overall electrical conductivity of PBHP-TAPT COF, after HCl/NH_3 cycles (error bar 15%)

Supplementary Table 3. Comparison of electronic properties and band gap of some semi-conducting materials.

Material	Type	Conductivity [S/m]	Band gap [eV]
Graphene[5]	Conductor	10^{2} - 10^{3}	-
Borocarbonitride[6]	Semiconductor	$\sim10^{5}$	1.0 – 3.9
MoS_2[7]	Semiconductor	$\sim10^{-4}$	1.2 – 1.9
TTF-COF (I-Doped)[8]	Semiconductor	$\sim10^{-4}$	-
Polyporphyrin-core COF[9]	Semiconductor	$\sim10^{-6}$	-
PBHP-TAPT COF	Semiconductor	$\sim10^{-8}$	2.3
Act. By HCl PBHP-TAPT COF	Semiconductor	$\sim10^{-6}$	2.0

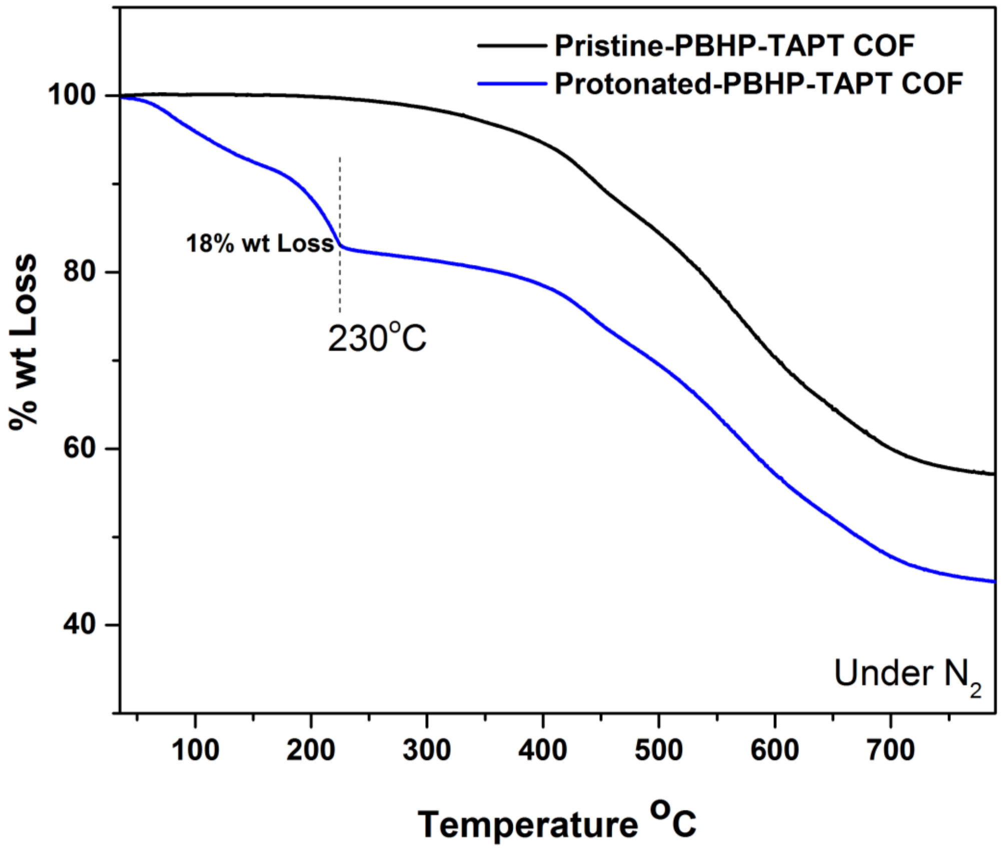

Supplementary Figure 25. Thermogravimetric analysis of pristine and protonated PBHP-TAPT COF, heating ramp at 10 ^{o}C/min, the amount of HCl gas adsorbed was estimated to be around 18%.

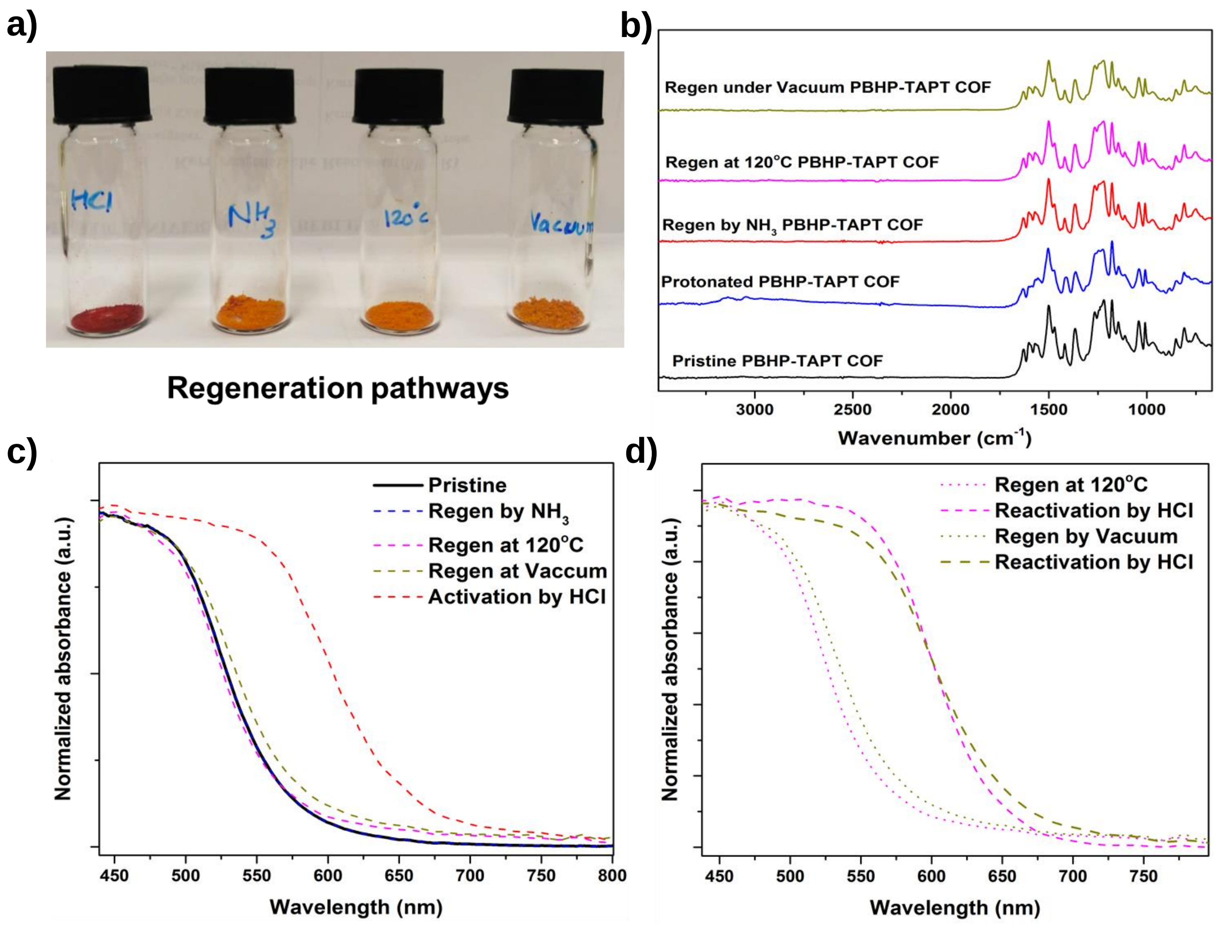

Supplementary Figure 26. a) Photographs indicating regeneration of protonated PBHP-TAPT COF left to right: protonated sample, regenerated via NH_3 treatment, regenerated via heat treatment at 120 ^oC, 60 min, and regenerated under high vacuum on Schlenk line for 24 h at RT, b) FTIR spectra of samples regenerated using different methods, c) UV-Vis diffusive reflectance spectra of regenerated PBHP-TAPT COF, and d) UV-Vis diffusive reflectance spectra of reactivated PHBP-TAPT COF by HCl vapours.

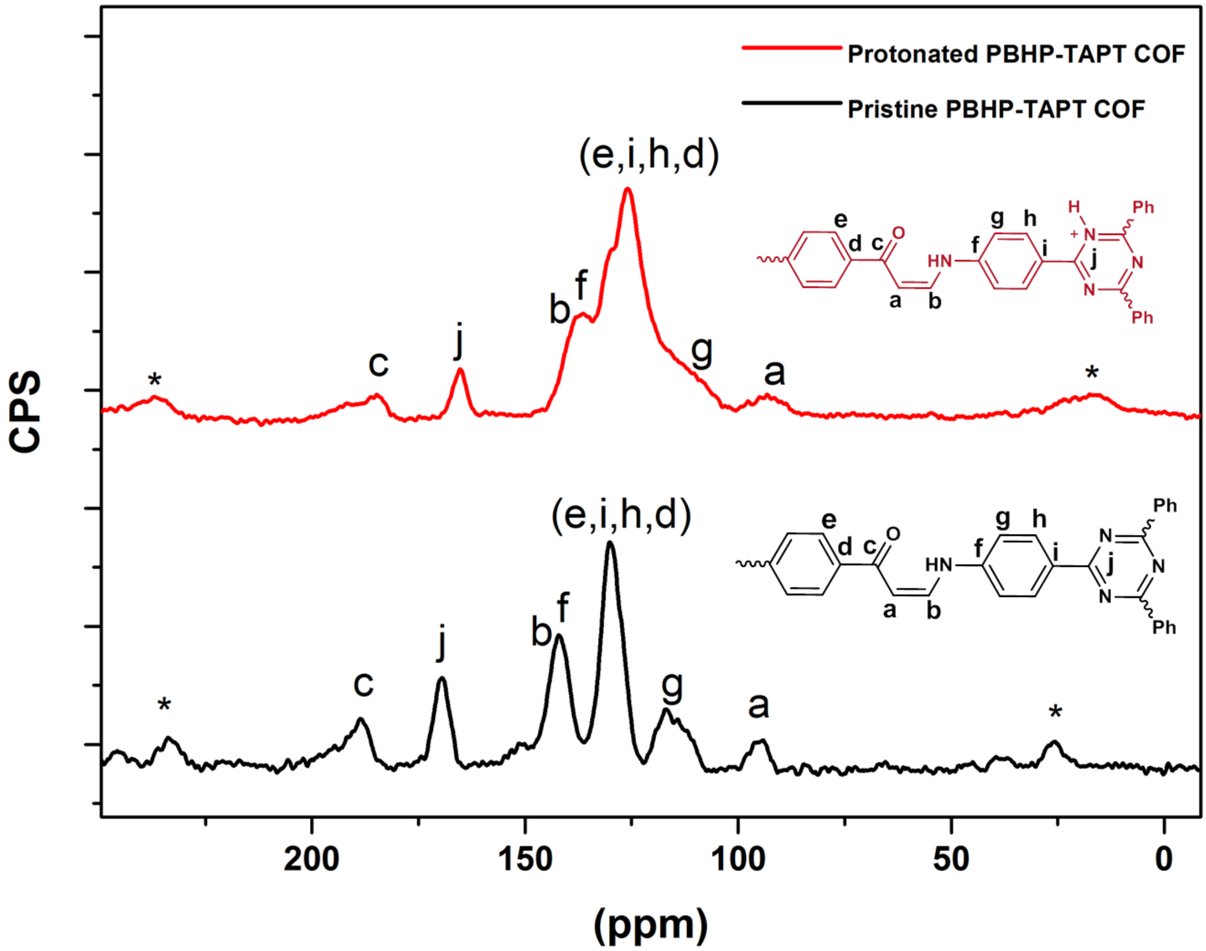

Supplementary Figure 27. Solid-state ^{13}C CP-MAS spectra of pristine and protonated PBHP-TAPT COF shown in black and in red, respectively.

Supplementary Note 3.

In order to further confirm the protonation site, we performed solid-state ^{13}C CP-MAS NMR on the HCl-activated PBHP-TAPT COF. 0.2 g of pristine sample was protonated using a steady stream of HCl gas for 10 s, as in all previous protonation experiments. ^{13}C CP-MAS solid-state NMR spectra of PBHP-TAPT COF were recorded in 3.2 mm rotors at 13 kHz, and protonated PBHP-TAPT COF spectra were obtained in 4 mm rotors at10 kHz. The ^{13}C signals were recorded for 12 h. It should be noted, that the 4 mm rotor was not absolutely air-tight, as we observed a color change of the protonated PBHP-TAPT COF from deep red to dark orange over the course of 12 h. Upon protonation, most peaks experience an upfield shift, with the notable exceptions of the aryl sp^2 carbon environment (e) and the keto-enamine carbon (a). This corresponds best to the scenario that the protonation site is preferentially at the ring-nitrogen of the triazine sub-unit (see Supplementary Figure 28).

Predicted ^{13}C NMR:

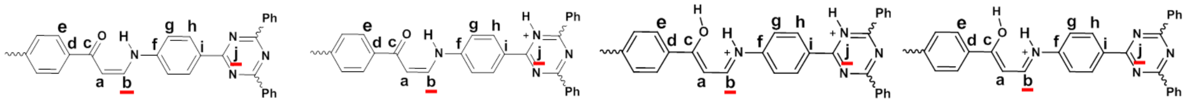

Carbon nuclei	Pristine Observed (ppm)	Protonated Observed (ppm)	Pristine Predicted [c] (ppm)	Triazine protonated [c] (ppm)	Triazine + KE protonated [c] (ppm)	Only KE protonated [c] (ppm)
a	95	93	94	94	78	78
b	147	140	146	146	157	157
c	189	186	189	189	177	177
d	139	131	137	137	135	135
e	129	130	128	128	127	127
f	140	135	139	139	143	143
g	114	112	114	114	125	125
h	128	126	128	128	128	127
i	127	121	124	116	125	134
j	179	165	172	162	162	172

Note: [c]= Predicted NMR signals
Blue = Downfield shift
Red = Upfield shift
Black = No change

Supplementary Figure 28. Observed and predicted ^{13}C signals of several protonation sites.

Supplementary Note 4.

The figure below shows the predicted chemical shifts of carbon signals upon protonation; all three different possibilities were considered and are compared in the table below: 1) protonation only at the triazine, 2) protonation at the triazine and keto-enamine bridge, and 3) protonation only at the KE. Based on the comparisons the ^{13}C NMR suggests that the triazine core is the preferred protonation site.

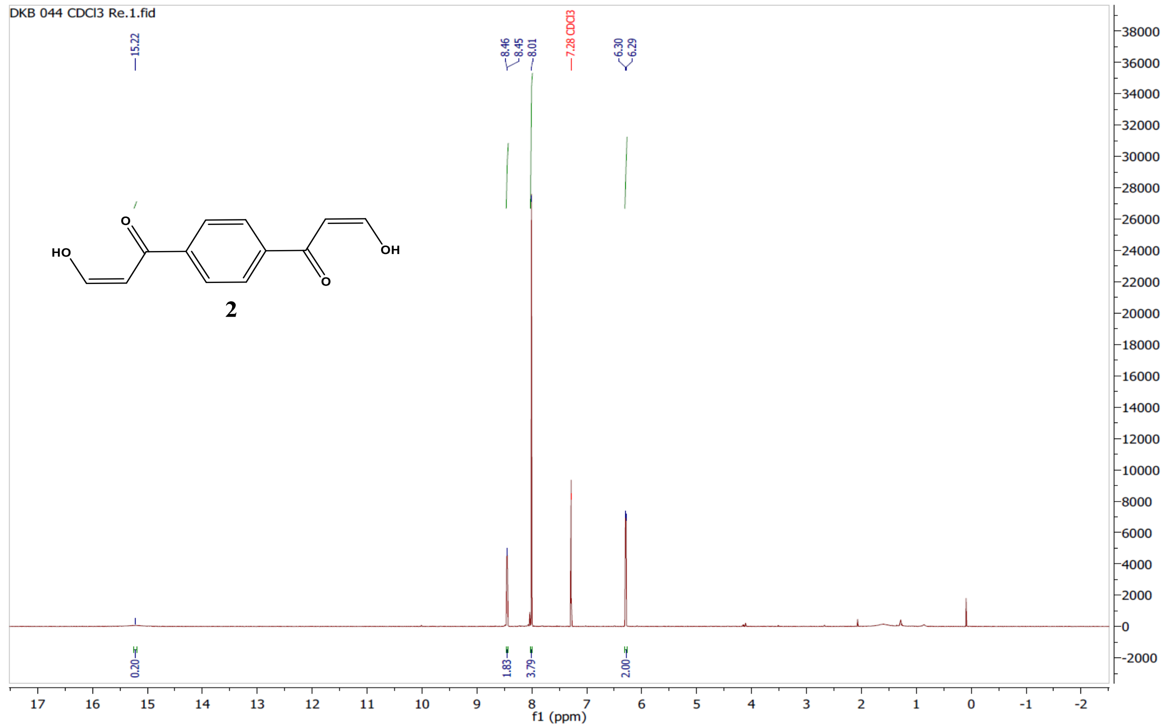

Supplementary Figure 29. ^{1}H NMR of (PBHP).

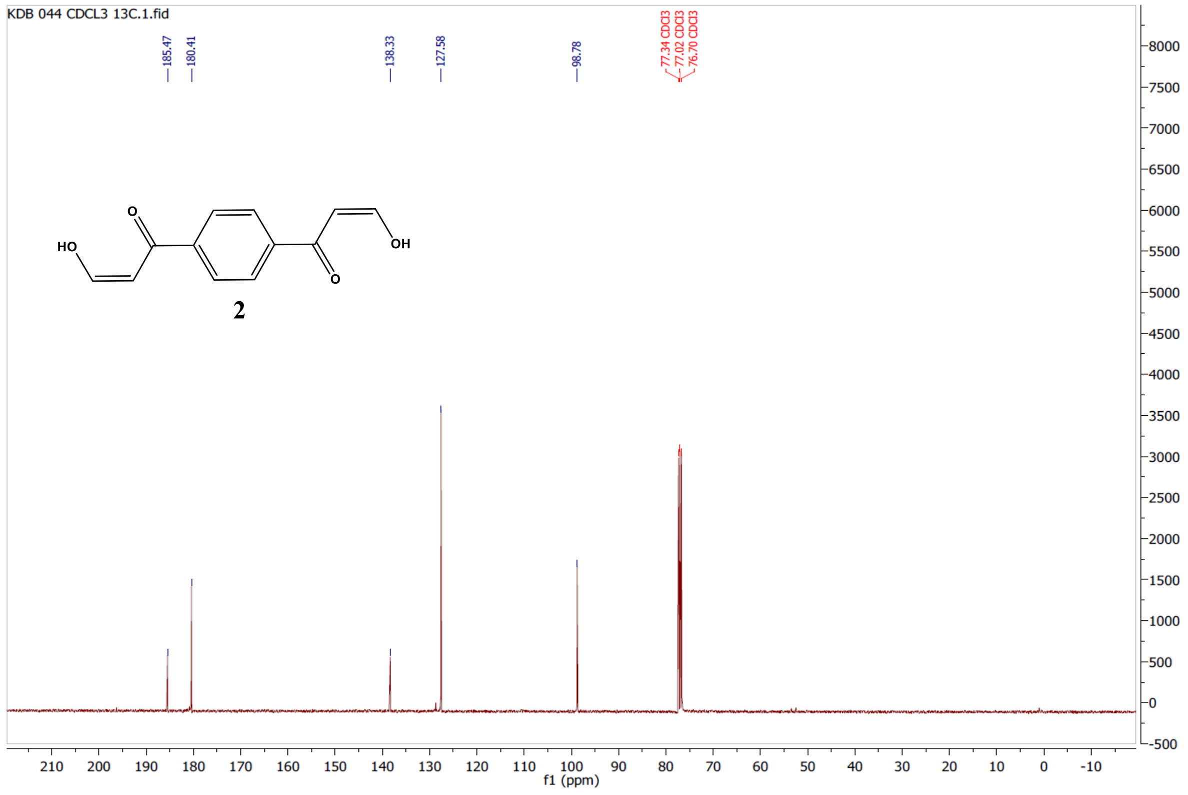

Supplementary Figure 30. ^{13}C NMR of (PBHP).

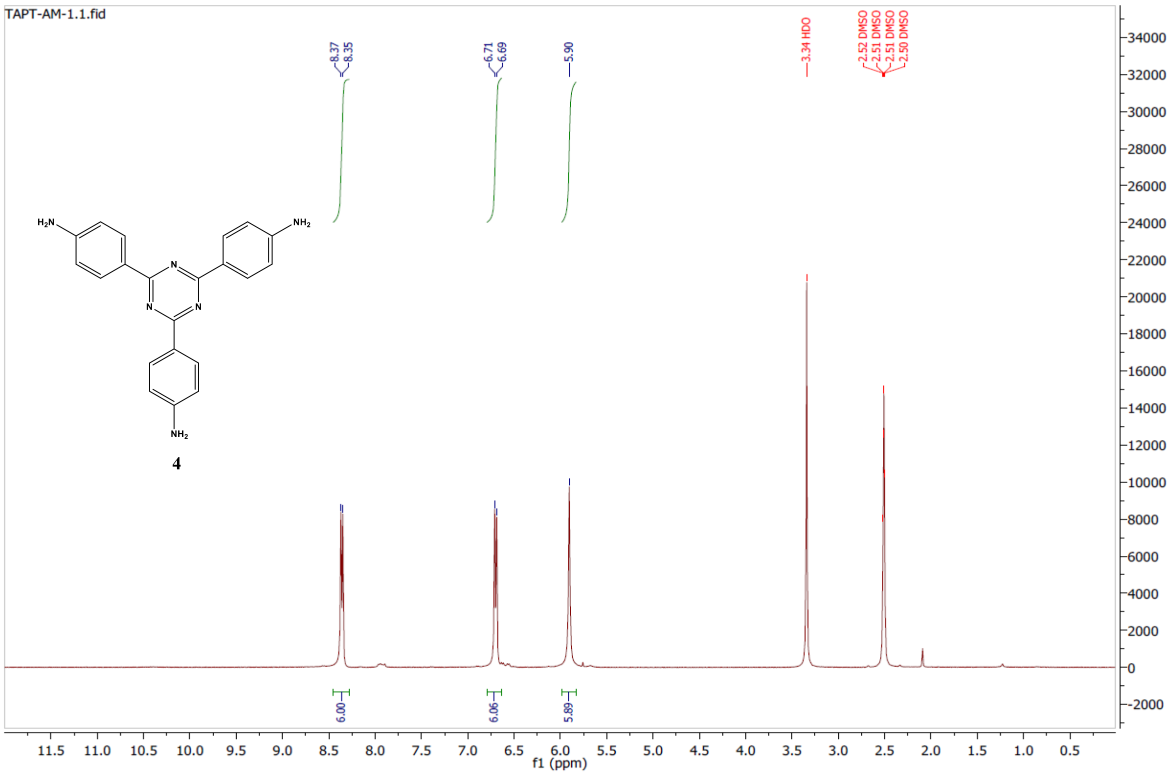

Supplementary Figure 31. ^{1}H NMR of (TAPT).

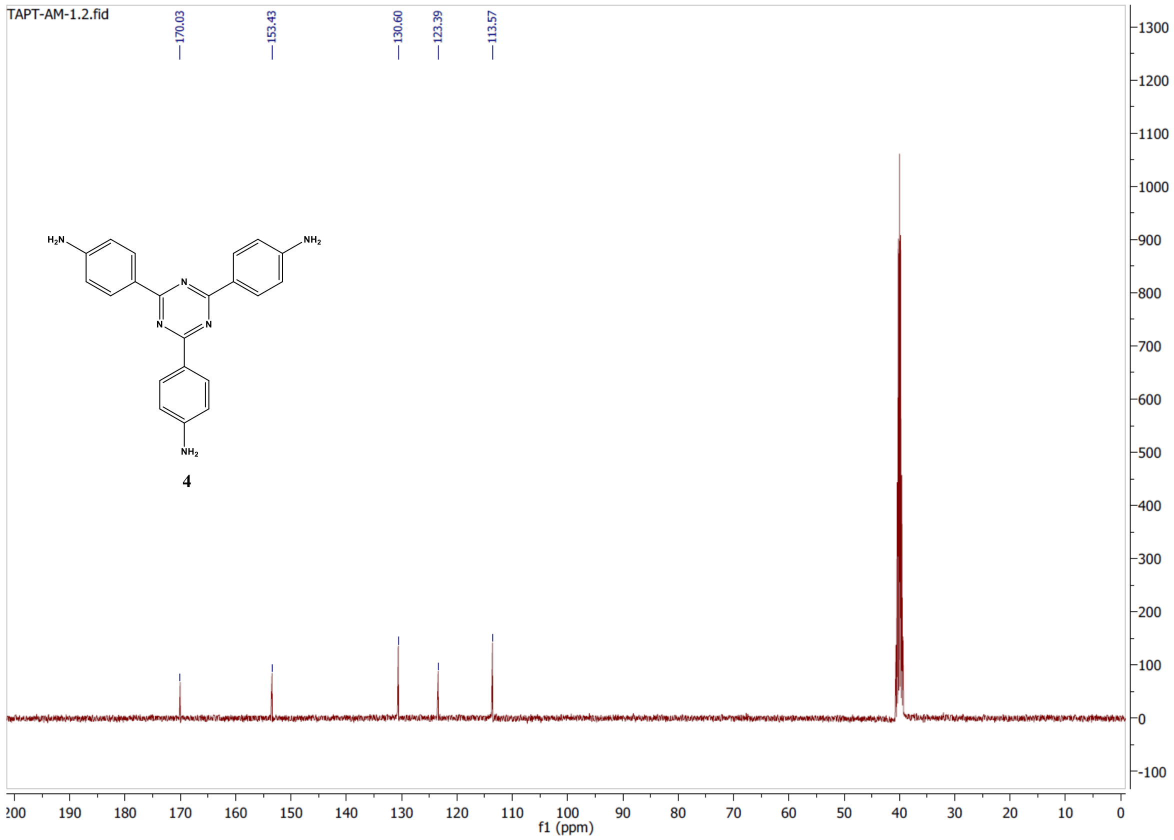

Supplementary Figure 32. ^{13}C NMR of (TAPT).

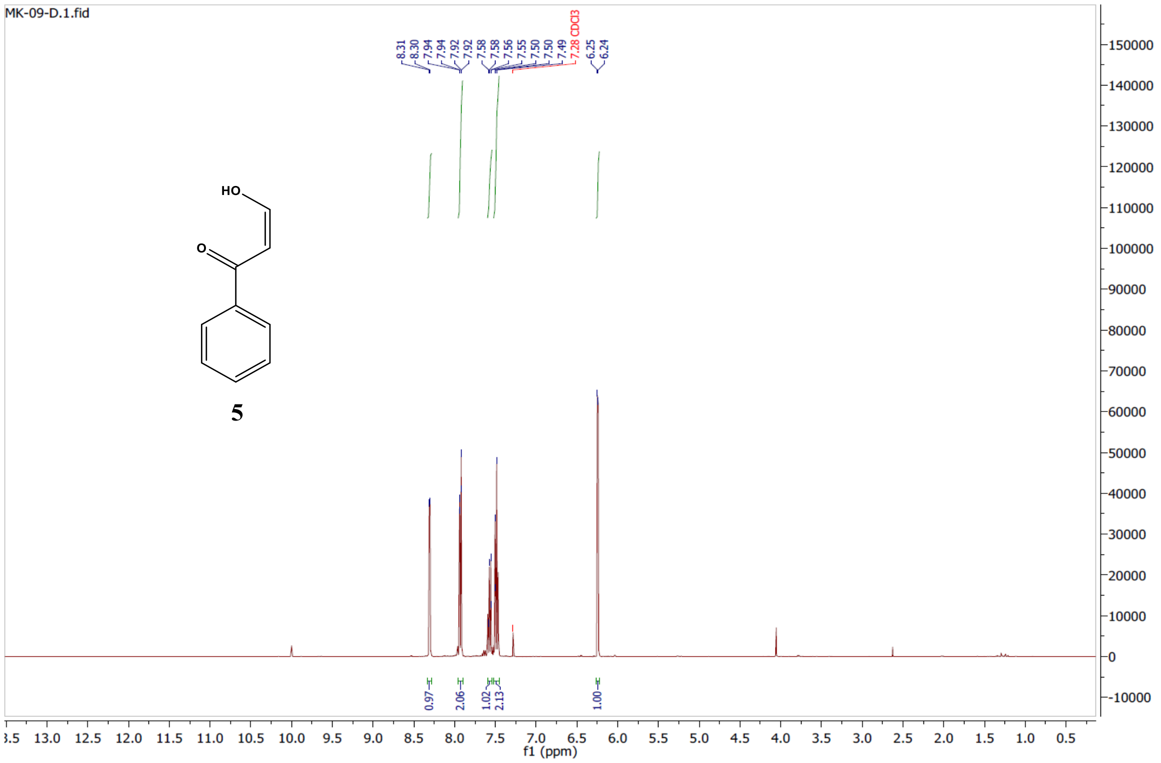

Supplementary Figure 33. ^{1}H NMR of (mono-keto-enol).

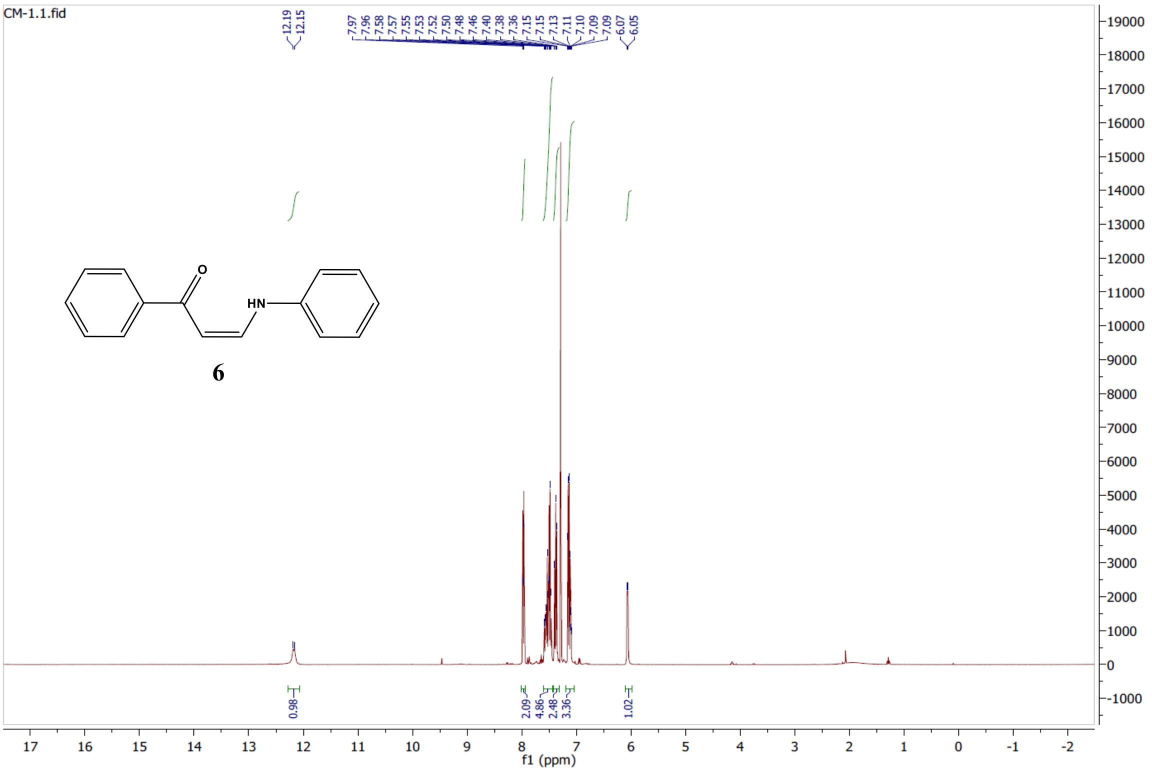

Supplementary Figure 34. ^{1}H NMR of (keto-enamine).

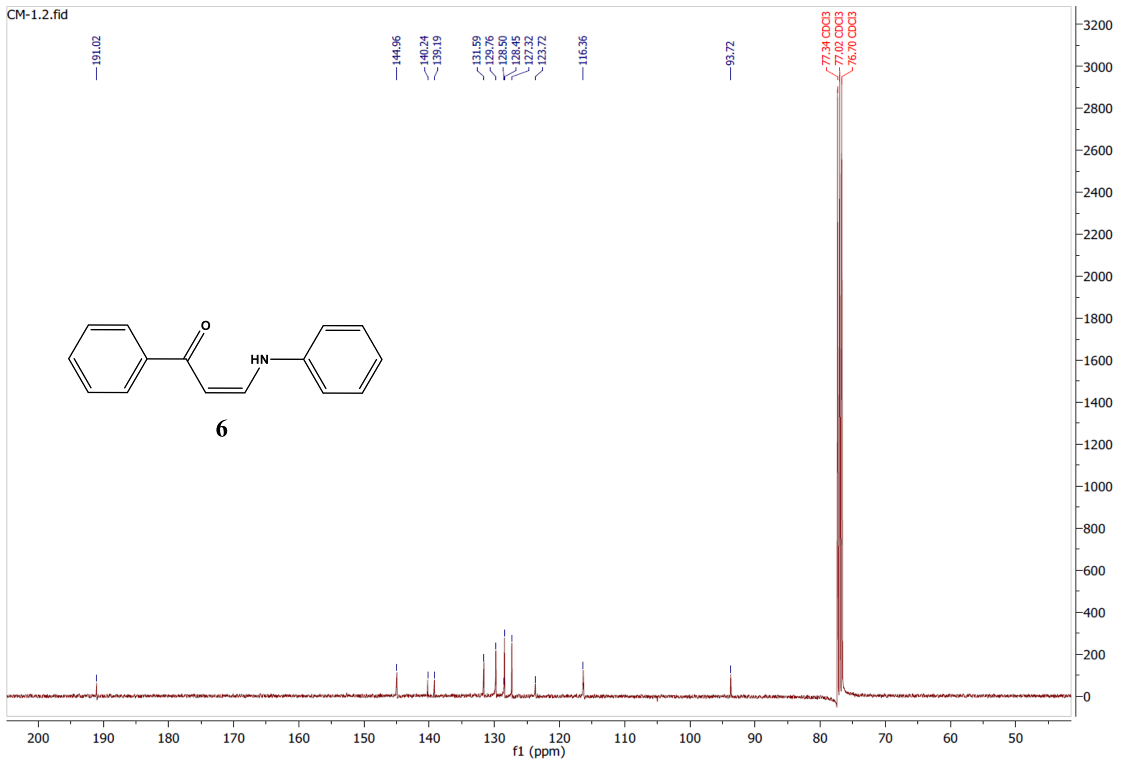

Supplementary Figure 35. ^{13}C NMR of (keto-enamine).

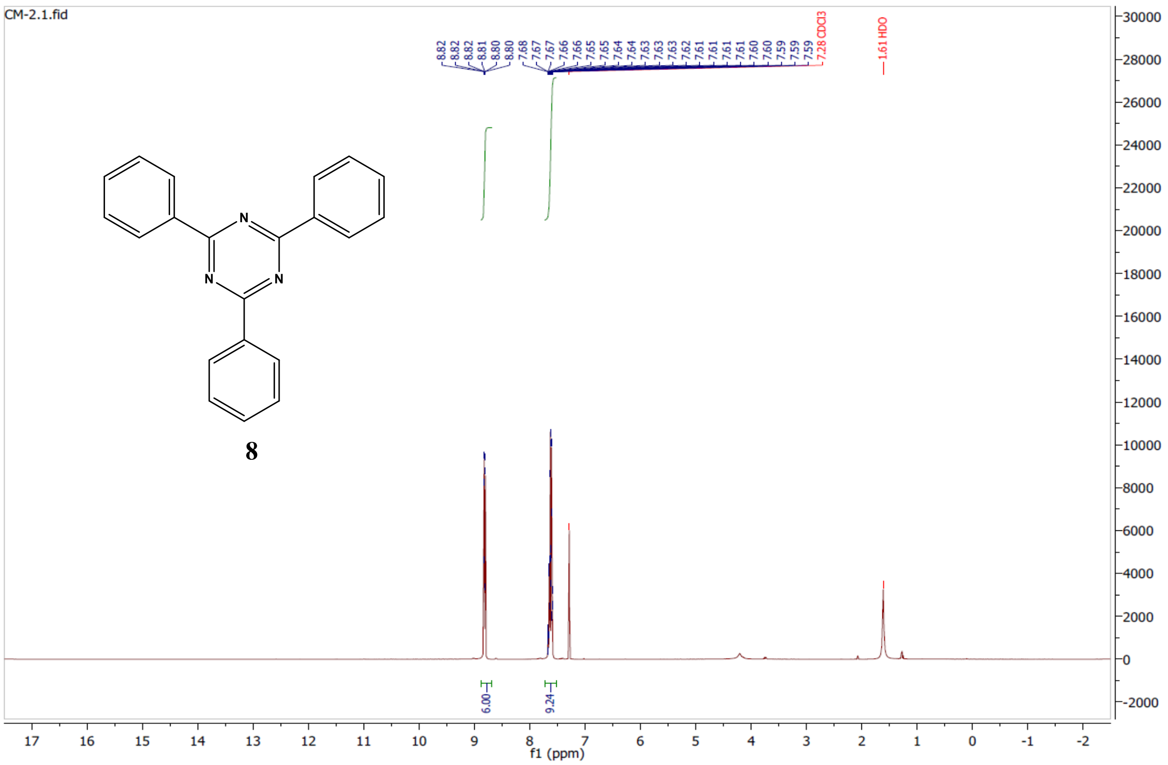

Supplementary Figure 36. ^{1}H NMR of (TPT).

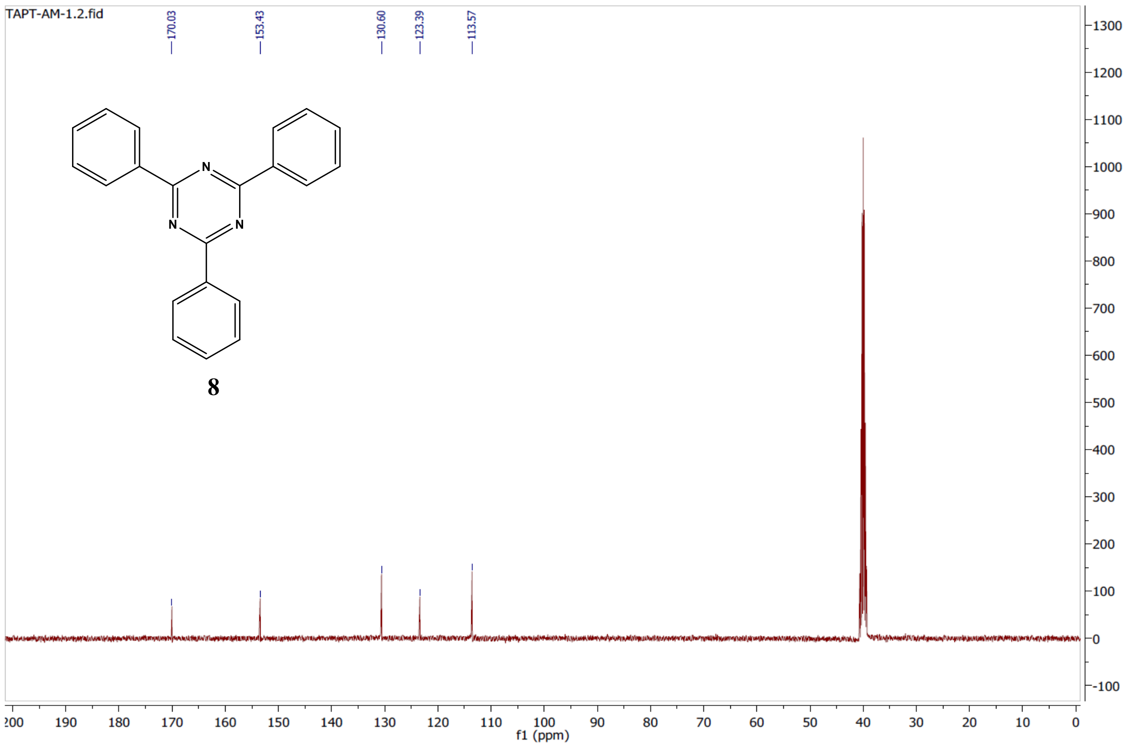

Supplementary Figure 37. ^{13}C NMR of (TPT).

Supplementary References.

1 Rao, M. R., Fang, Y., De Feyter, S. & Perepichka, D. F. Conjugated covalent organic frameworks via michael addition–elimination. *J. Am. Chem. Soc.* 139, 2421-2427 (2017).

2 Gomes, R., Bhanja, P. & Bhaumik, A. A triazine-based covalent organic polymer for efficient CO 2 adsorption. *Chemical Communications* 51, 10050-10053 (2015).

3 Li, M., Fang, D., Geng, F. & Dai, X. Silver-catalyzed efficient synthesis of enaminones from propargyl alcohols and amines. *Tetrahedron Letters* 58, 4747-4749 (2017).

4 Isfahani, A. L. *et al.* Palladium nanoparticles immobilized on nano-silica triazine dendritic polymer (Pdnp-nSTDP): An efficient and reusable catalyst for Suzuki–Miyaura cross-coupling and Heck reactions. *Advanced Synthesis & Catalysis* 355, 957-972 (2013).

5 Marinho, B., Ghislandi, M., Tkalya, E. & Koning, C. E. Electrical conductivity of compacts of graphene, multi-wall carbon nanotubes, carbon black, and graphite powder. *Powder Technology* 221, 351-358 (2012).

6 Ci, L. *et al.* Atomic layers of hybridized boron nitride and graphene domains. *Nature materials* 9, 430 (2010).

7 Van Der Zande, A. M. *et al.* Grains and grain boundaries in highly crystalline monolayer molybdenum disulphide. *Nature materials* 12, 554 (2013).

8 Ding, H. *et al.* A Tetrathiafulvalene-Based Electroactive Covalent Organic Framework. *Chem. Eur. J.* 20, 14614-14618 (2014).

9 Yang, H. *et al.* High conductive two-dimensional covalent organic framework for lithium storage with large capacity. *ACS applied materials & interfaces* 8, 5366-5375 (2016).
