# Supplementary material for: Real-time optical and electronic sensing with a β-amino enone linked, triazine-containing 2D covalent organic framework
Source: Nat Commun. 2019 Jul 19;10:3228. doi: 10.1038/s41467-019-11264-z (PMC6642192; doi:10.1038/s41467-019-11264-z)
Supplement: Supplementary file 3 — Description of Additional Supplementary Files [file 41467_2019_11264_MOESM3_ESM.pdf]

Description of Additional Supplementary Files

File name: Supplementary Movie 1

Description: A vial containing PBHP-TAPT COF is subjected to HCl (g) and subsequently to NH_3 (g). The visual response is recorded in real-time (x1). The experimental set-up is detailed as a sketch within the video.

File name: Supplementary Movie 2

Description: PBHP-TAPT COF powder is exposed to HCl (g) and subsequently to NH_3 (g) under a UV lamp (λ = 365 nm). The visual, fluorescence response is recorded in real-time (x1). The experimental set-up is detailed as a sketch within the video.
